# Will the inclusion of AI anchors enhance the operational performance of live streaming e-commerce supply chains?

**DOI:** 10.1371/journal.pone.0321995

**Published:** 2025-06-17

**Authors:** Xinyan Yan, Qihui Lu, Di Xiao

**Affiliations:** 1 Modern Business Research Center of Zhejiang Gongshang University, Hangzhou, China; 2 Inamori Business School, Zhejiang Gongshang University, Hangzhou, China; 3 School of Business Administration (MBA), Zhejiang Gongshang University, Hangzhou, China; 4 Enterprise Digital Intelligence and Business Analysis Research Center, Zhejiang Gongshang University, Hangzhou, China; Sri Sivasubramaniya Nadar College of Engineering, INDIA

## Abstract

With the rapid growth of the live streaming e-commerce market, traditional live streaming models are encountering mounting challenges, whereas the advent of artificial intelligence (AI) technology has breathed new life into live streaming. This paper delves into the role of the AI anchor and model selection in scenarios both with and without the involvement of the Key Opinion Leader (KOL). Specifically, the AI anchor, whether invested in by the brand or the live streaming platform, is integrated into the regular anchor model (Model NN) and the hybrid model combining the KOL and the regular anchor (Model NK). A series of Stackelberg game models are formulated to compare the sales effort level, AI intelligence level, anchor influence, and the proportion of live streaming time on live streaming e-commerce. The findings suggest that the AI anchor can effectively substitute for the human anchor under certain conditions, particularly when the brand controls the AI intelligence level. To further explore the impact of the AI anchor on decision-making and profits among supply chain participants, the paper offers a thorough analysis of decisions and profits in scenarios both with and without KOL involvement. The results disclose that, under suitable conditions, the incorporation of the AI anchor can substantially enhance the operational efficiency of live streaming e-commerce supply chains, leading to increased profitability for the brand. Notably, the allocation of live streaming time between different anchors in hybrid models significantly influences the profitability of supply chain members. This discovery provides crucial insights for brands in devising live streaming strategies.

## 1 Introduction

### 1.1 Background and motivation

In recent years, with the widespread adoption of digital media and the rapid growth of e-commerce, the live streaming e-commerce market has been exhibiting exponential growth. Consumers increasingly prefer live streaming e-commerce as their shopping method of choice [1–3]. As of 2023, live streaming e-commerce sales in the United States had reached 50 billion, with projections indicating a 36% increase in real-time online shopping sales by 2026, accounting for over 5% of all e-commerce sales in North America. Live streaming e-commerce not only stimulates consumer demand through live streaming channels but also positively influences the e-commerce supply chain [4]. According to the definition provided by The Institute for Supply Management (ISM), a supply chain is a value-added business network comprising enterprises and departments involved in material acquisition, processing, and the delivery of finished products to end-users. This network is centered around a core enterprise and achieves its functionality through the management of information flow, logistics, and financial flow. In the context of live streaming e-commerce, the supply chain refers to a model where brand owners utilize anchors—including regular anchors, KOLs, and AI anchors—to interact with consumers in real-time on live streaming e-commerce platforms, facilitating the introduction and sale of products and services [5].

With the rise of KOLs, brands increasingly value their market influence, frequently inviting KOLs to test products and entrusting them with product sales through live streaming. However, as the collaboration between the brand and KOL increases, the brand gradually realizes that selling through KOL not only entails paying high commission fees and fixed pit fees, but also demands significant product discounts. Additionally, this approach often fails to bring stable traffic to the store [6], resulting in minimal profits or even losses. Despite this, the brand is still reluctant to abandon the vast live streaming e-commerce market, leading to the exploration of other live streaming formats, such as sales by regular anchors on live-streaming platforms. Since 2022, major live streaming platforms have actively supported the regular anchor. Tao Fang, General Manager of the Taobao Live Business Unit, declared that Taobao is intentionally fostering regular anchors and providing “unprecedented" support in terms of algorithms, product selection, traffic, and bonuses to promote the growth of new anchors and the cultivation of existing ones.

Currently, many brands prefer using the regular anchor for live streaming, primarily due to cost-effectiveness. The regular anchor typically has an in-depth understanding of store products and can effectively convey the core value of the product. But the brand also hopes that the anchor can bring followers to boost sales. Therefore, the brand will still invite KOL for live streaming to enhance brand influence. For example, on December 18, 2023, Walmart invited several KOLs, including Michael Le and Andrea Espaada, to sell products through its live stream on TikTok. As a result, two common live streaming models have emerged: the regular anchor model (Model NN) and the mixed model featuring both regular anchor and KOL (Model NK). However, in both the models NN and NK, the sales efforts of the regular anchor and KOL are self-determined, making it difficult for the brand to exercise control. Moreover, although KOL can bring more traffic, the brand not only loses control over retail pricing but also incurs higher commission fees, which decreases profit margins. This highlights that traditional live streaming models can no longer fully meet the needs of the brand.

The emergence of artificial intelligence has introduced new opportunities for live streaming. Some companies began employing AI anchors to replace human anchors for product presentations and intelligent interactions. For example, companies such as Microsoft, TikTok, and iFLYTEK have launched AI anchor-related products. On April 16, 2024, JD.com founder, Richard Liu, debuted the “Sales Liu AI Digital Human" during a live stream, attracting more than 20 million viewers in one hour, with total sales exceeding 50 million yuan by the end of the stream. According to a report by The Org, the performance of the AI anchor is almost comparable to that of a human anchor, indicating that AI live streaming can potentially replace human anchors to a certain extent [7]. The primary members of the live streaming e-commerce supply chain include the live streaming platform, brands, regular anchors, and KOLs. However, AI anchors have varying impacts on different members of the supply chain. First, for regular anchors, AI anchors can operate 24/7, providing uninterrupted live streaming services to meet consumer demands at any time [8]. This characteristic, to some extent, compresses the broadcasting time and audience base of regular anchors, and it may gradually lead to their partial replacement. Second, for KOLs, AI anchors, devoid of human emotions, remain patient and efficient when addressing frequent and repetitive questions, without exhibiting fatigue or annoyance [9]. This allows KOLs to allocate more time and energy to creating high-quality content. However, this also imposes an unprecedented sense of crisis on KOLs, as they face the risk of losing their audience to highly influential AI anchors. Moreover, for brands, AI anchors can assist companies in reducing costs, enhancing operational efficiency, and improving overall productivity [10]. Ding Wen, the executive president of Swisse China, believes that the AI anchor can achieve high output with low input, playing a key role in driving sales. International brands such as Philips, L’Oréal, and Unilever, along with domestic brands like Perfect Diary, Chando, and Florasis, have already started using the AI anchor in their official live room [11]. It is worth noting that the homogeneity of AI anchor content may weaken consumer brand loyalty. For live streaming platforms, the introduction of AI anchors enriches the content ecosystem. Platforms can leverage the consistent performances of AI anchors to attract more brands and viewers, thereby fostering the prosperity of their ecosystems. Nonetheless, excessive reliance on AI anchors may undermine the support and development of regular anchors and KOLs, potentially affecting the platform’s long-term growth and diversity.

From the brand’s perspective, there are fundamental differences between the regular anchor and KOL. First, in terms of traffic generation, the regular anchor struggles to generate the ideal sales performance without relying on external traffic, while KOL has a clear advantage in enhancing store influence and drawing more viewers to live streams. Second, regarding pricing power, the brand retains control over product pricing when working with the relatively weaker regular anchor, whereas KOL, with its greater influence, often commands more say in pricing, potentially resulting in the brand losing control over pricing decisions. Therefore, the brand needs a new force, and the introduction of the AI anchor offers an innovative option for live streaming on live streaming platforms. Through the introduction of the AI anchor, the brand can regain control over sales efforts and product pricing, thereby increasing profitability. A comparison between the regular anchor, KOL, and the AI anchor is provided in Table 1.

**Table 1:** List of research models.

	Regular anchor	KOL	AI anchor
Pricing Power	Similar to regular employees, no pricing power.	Strong bargaining power, has pricing authorit.	As an introduction of new technology, no pricing power.
Commission Rate	Relatively low.	High, plus fixed pit fees.	1.Brand investment: No commission rate, but with investment costs; 2.live Streaming platform investment: No commission rate or investment costs for brand using AI anchor.
Brand Control over anchor	No control.	No control.	When brand invest, they can control the intelligence level of the AI anchor.
Anchor Influence	Average effectiveness.	Best effectiveness compared to other anchor.	Average effectiveness.

### 1.2 Research questions and major findings

Based on the above observations and discussions, we identify significant differences between the regular anchor and the KOL in terms of live stream traffic and decision-making authority. Furthermore, the effects of introducing the AI anchor in these two contexts vary. For the current live streaming models (i.e., Models NN and NK), this study aims to address the following four research questions:

(i) Under what conditions is the introduction of AI anchors more advantageous for supply chain members?

(ii) How do the attractiveness of different anchors and the allocation of live streaming time impact supply chain members under various live streaming models?

(iii) What effects do AI anchors funded by brands versus those funded by live streaming platforms (hereafter referred to as AI anchors with different investment sources) have on supply chain members?

(iv) How should brands make decisions to ensure the robustness of the live streaming e-commerce supply chain?

The main findings of this study are summarized as follows. First, the introduction of AI anchors benefits brands, irrespective of KOL involvement or whether the AI anchor is funded by the brand or the live streaming platform. Notably, the inclusion of AI anchors negatively impacts other live streaming participants within the ecosystem, namely regular anchors and KOLs. Second, the greater the influence of AI anchors, the more advantageous it is for the brand, regardless of KOL involvement or the source of AI anchor funding. However, in scenarios involving KOLs, when the influence of the KOL exceeds a certain threshold, the brand’s profit decreases as the KOL’s influence increases. In scenarios without KOL participation, increasing the proportion of live streaming time allocated to regular anchors may weaken the brand’s motivation to adopt AI anchors. Conversely, in scenarios with KOL participation, increasing the proportion of live streaming time allocated to KOLs effectively decreases the likelihood of the brand adopting AI anchors. Third, brands derive more benefits from introducing AI anchors that they fund themselves, regardless of KOL involvement. For human anchors, in scenarios without KOL participation, brand-funded AI anchors are more advantageous for regular anchors. However, in scenarios with KOLs, AI anchors invested in by brand owners or live streaming platforms have the same impact on other supply chain members. Fourth, if AI anchors are not introduced, in scenarios without KOLs, a “win-win” for supply chain members can only be achieved when regular anchors have a high proportion of live streaming time and exhibit strong influence. In scenarios involving KOLs, a “triple-win” can only be achieved when regular anchors have a low proportion of live streaming time and both regular anchors and KOLs have strong influence. If the brand opts to introduce AI anchors, those funded by the brand benefit supply chain members, whether or not KOLs are involved, facilitating either a “win-win” or “triple-win” outcome.

### 1.3 Contribution statements and organization

This study investigates the impacts of anchor effort levels, live streaming duration, AI anchor intelligence levels, and different investment models on live streaming e-commerce. The contributions of this paper are summarized as follows:

1. Most existing research on live streaming e-commerce focuses on the influence of human anchors on market demand. Only a limited number of studies consider AI anchors, and none have examined the effects of AI intelligence levels and investment models. This research contributes to the literature by developing a game-theoretical model to quantitatively analyze the impacts of AI anchor intelligence levels and investment models on the live streaming e-commerce supply chain.

2. We quantitatively analyze a multi-anchor hybrid live streaming model where regular anchors, KOLs, and AI anchors coexist on the platform. This study identifies the effects of live streaming duration allocation on the supply chain under scenarios where brands or live streaming platforms invest in AI anchors. To the best of our knowledge, this is the first study to consider a hybrid model involving three types of anchors on live streaming platforms. Furthermore, it is also the first to explore the allocation of streaming duration in the context of live streaming e-commerce.

3. This research examines the value of anchor effort levels and AI anchor intelligence levels under scenarios with and without KOL participation. Our results demonstrate that brands benefit from AI anchors regardless of whether KOLs are involved. However, the situation for human anchors may deteriorate. While most existing studies focus either on anchor effort levels or AI intelligence levels, few integrate these aspects. Thus, this study provides managerial insights into the introduction of AI anchors in the live streaming e-commerce context.

The structure of this paper is as follows: Sect 2 provides a brief review of the relevant literature and highlights the uniqueness of this study, distinguishing it from previous research. Sect 3 outlines the basic assumptions and constructs the research model. Sects 4 and 5 focus on scenarios without KOL involvement and with KOL involvement, respectively, using the Model NN and NK as benchmarks. These sections delve into the optimal decisions of supply chain members when the AI anchor with different investors is introduced, followed by a comparative analysis (the research model is shown in Table 2). Finally, Sect 6 summarizes the study and offers prospects for future research directions.

**Table 2:** List of research models.

		Introducing AI Anchor	
Section	No AI Anchor (N)	Brand bear investment cost (B)	live streaming platform bear investment cost (P)
Section 4. No KOL	Model NN	Model BN	Model PN
Section 5. With KOL	Model NK	Model BK	Model PK

## 2 Literature review

This paper reviews the relevant literature on live streaming e-commerce, AI-driven operations management, and sales effort, aiming to provide a comprehensive overview of current research trends and a solid theoretical foundation for future studies.

### 2.1 Live streaming e-commerce

In recent years, the rapid development of live streaming e-commerce has attracted significant scholarly attention. Many studies have focused on the choice of sales strategies and their impact on profitability and market competitiveness. Liu *et al*. [12] explored whether and when e-commerce platforms should introduce live streaming channels. Huang *et al*. [13] examines the impacts and strategies of live stream channel introduction for competing retailers. Hao and Yang [14] analyzed the application of resale and agency sales models in live streaming e-commerce. Furthermore, Li *et al*. [15] examined the optimal sales strategies for omnichannel manufacturers under the trend of adopting live streaming demonstrations. Li *et al*. [16] evaluated online retailers’ live streaming sales strategies under spillover effects, as well as their implications for consumer surplus and social welfare. Meanwhile, scholars have conducted in-depth research on the mechanisms of live streaming e-commerce, Ji *et al*. [17] examined sales model selection under different price discount strategies. Cui *et al*. [18] investigated the role of live streaming e-commerce in reducing uncertainty regarding product suitability. Lu and Duan [19] explored live streaming strategies for vertically differentiated companies selling products of varying quality. Some scholars have also approached the topic from the perspective of the anchor, with particular attention given to the role of KOL in live streaming e-commerce. Meng *et al*. [20] explored the economic value of KOL, specifically analyzing the impact of KOL performance on consumer purchase intention. Zhang *et al*. [21] compared the merchant live streaming and KOL live streaming models on retail platforms. Liu and Wang [22] focused on whether companies should opt for one-time or multiple sales through KOL on live streaming platforms. Niu *et al*. [23] examined the effectiveness of using KOL to promote products through live streaming channels. Zhang *et al*. [24] explored the selection of merchant live streaming, KOL live streaming, and hybrid models. Chen *et al*. [6] further examined the impact of KOL sales effort and personal influence on the coordination, pricing, and sales decisions of dual-channel supply chains. Ye *et al*. [25] analyzed sellers’ preferences for different types of influencers and their pricing strategies from a live streaming perspective. Lu *et al*. [26] examined the optimal strategies for retailers and manufacturers who collaborate with anchors to establish live streaming sales channels by sharing demand information. Some scholars have also studied the impact of AI anchors on live streaming. Niu *et al*. [27] compared the performance of AI anchors and KOL in cross-border operations, and Xu *et al*. [9] considered whether live streaming platforms should introduce AI anchors.

### 2.2 Operation management based on AI

This study also involves the introduction of AI anchor, thereby closely aligning with the literature on AI in the field of operational management. With the advancement of AI technology, virtual influencers have gained increasing prominence on social media platform and are increasingly participating in product promotions [28]. Scholars have identified certain advantages of AI anchor over human anchor, as highlighted by Miao *et al*. [29], who noted that the primary advantage of virtual anchor lies in the controllability of their speech and behavior, which contributes to mitigating negative incidents that may harm the reputation of brand or live streaming platform. Peng *et al*. [30] investigated consumer acceptance of AI services in different contexts. Li *et al*. [31] and Zhou *et al*. [32] delved into the fundamental differences between human and virtual influencers. Additionally, scholars have examined the impact of AI anchor on customer purchase intention in the context of live streaming e-commerce, with Niu *et al*. [27] and Xu *et al*. [9] studying supply chains comprising manufacturers and live streaming platform and exploring the influence of AI anchor on these entities. Further research has explored the impact of anchor types (AI vs. human) and characteristics of virtual anchor (such as likability, vitality, responsiveness, and sociability) on consumer purchase intention, social presence, telepresence, and experiential value [33, 34]. Some scholars have also studied the technical characteristics of VLSP (anthropomorphism and media richness) and their effects on customers’ purchase intentions, as well as how trust in anchors influences purchase intentions in live streaming e-commerce [8, 35].

### 2.3 Sales effort

This study is also closely related to the literature on sales effort. A substantial body of research has been devoted to pricing and sales effort decisions. For example, Wang and Song [36] discussed pricing strategies in a dual-channel supply chain considering sales effort and demand uncertainty. Hu *et al*. [37] examined pricing and sales effort decisions in a CLSC with different sales formats (no sales, retailer sales, and OEM sales). Zhang *et al*. [38] studied product pricing, carbon reduction, and sales effort decisions in a retailer-led two-echelon low-carbon supply chain comprising a manufacturer and a retailer. Datta *et al*. [39] analyzed optimal selling prices, sales effort, and profits under stochastic demand in a dual-channel (online and offline) setting. Additionally, research on dual-channel supply chains has explored the impact of sales efforts from various perspectives. Sha and Zheng [40] investigated how offline retailers’ sales efforts affect demand within decentralized dual-channel supply chains comprising independent manufacturers, offline retailers, and online retailers. Building on this foundation, Sun and Liu [41] examined the role of sales efforts in dual-channel supply chains that include brick-and-mortar retailers, manufacturers, and manufacturer-owned e-commerce platform stores. Other scholars have approached the topic from different angles. For instance, Taylor [42] explored the influence of sales efforts on supply chain coordination through the lens of channel rebates. In a different context, Sane *et al*. [43] discussed the interplay between sales efforts and recycling quantities in manufacturer-dominated closed-loop supply chains. More recently, Wu *et al*. [44] analyzed how asymmetrical sales efforts by retailers, under manufacturer-supported strategies, impact operational decisions and the profits of channel members.

### 2.4 Research gap

In this subsection, we summarized the research gap between our study and the related literature and further highlighted our contributions. By synthesizing the main points of the current literature, it can be observed that while Zhang *et al*. [21], Chen *et al*. [6] and Zhang *et al*. [24] consider the KOL and its sales efforts, they do not address the issue of the AI anchor and its intelligence level. Similarly, Niu *et al*. [27] investigate the influence of the AI anchor’s intelligence level and the KOL on cross-border e-commerce, and Xu *et al*. [9] explore the relationship between sales efforts and the AI anchor. However, neither study examined the influence of anchor impact on live streaming sales. Furthermore, none of these studies addressed the proportion of live streaming time, which is a critical factor in practice. Given the limited total live streaming time and the common occurrence of mixed-anchor live streaming scenarios, we believe it is essential to analyze the effect of the proportion of live streaming time allocated to each anchor on live streaming sales. Our research builds upon this foundation by incorporating the regular anchor, the KOL, and the AI anchor into a unified analytical framework, comprehensively considering the degree of sales efforts by the anchor, the intelligence level of the AI anchor, and the influence of the anchor, while also introducing the proportion of live streaming time by the anchor, yielding some important and interesting findings. Table 3 summarizes the key distinctions between our research and existing studies.

**Table 3:** Differences between this paper and most related papers.

	Sales effort	Intelligence level	KOL	AI	Anchor influence	Percentage of live broadcast time of the anchor
Zhang *et al*. [21]	√	×	√	×	×	×
Chen *et al*. [6]	√	×	√	×	√	×
Zhang *et al*. [24]	√	×	√	×	√	×
Niu *et al*. [27]	×	√	√	√	×	×
Xu *et al*. [9]	√	×	×	√	×	×
Our paper	√	√	√	√	√	√

## 3 Model

**Fig 1:**
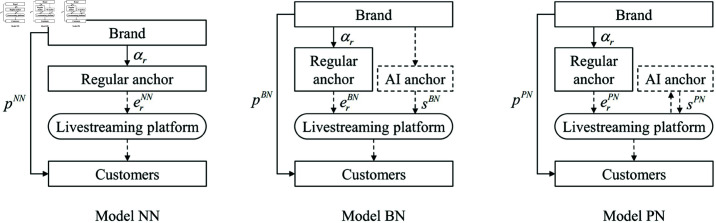
Supply chain structures without KOL involvement.

**Fig 2:**
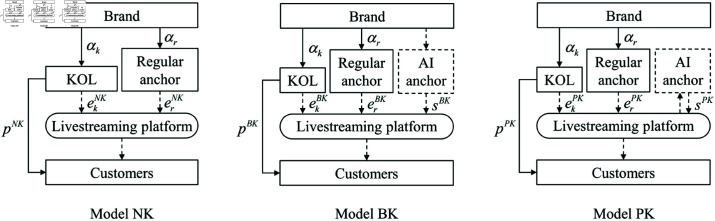
Supply chain structures with KOL involvement.

We explore a live streaming sales framework that involves a partnership between a brand and a live streaming platform, wherein the brand operates a dedicated live streaming channel on the platform. The primary configurations of live streaming are divided into two fundamental models: the regular anchor mode, which does not include KOL participation (labeled as *NN*), and the mixed mode that incorporates both regular anchors and KOLs (labeled as *NK*). Building upon this framework, we examine an AI anchor model, which is financially supported either by the brand or the live streaming platform, leading to four hybrid models: the combined mode of a regular anchor and an AI anchor with the brand covering investment costs (labeled as *BN*), the combined mode of a regular anchor and an AI anchor with the platform covering investment costs (labeled as *PN*), the combined mode including a regular anchor, a KOL, and an AI anchor with the brand responsible for investment costs (labeled as *BK*), and the combined mode including a regular anchor, a KOL, and an AI anchor with the platform responsible for investment costs (labeled as *PK*). Figs 1 and 2 illustrate the supply chain configurations with and without KOL involvement, respectively.

Drawing upon relevant literature in marketing and operations management [21, 24], we define the demand functions for the regular anchor (denoted by subscript *r*) and the KOL (denoted by subscript *k*) as follows: $d_{j}^{i}=t_{j}^{i}(d_{0}+\lambda_{j}e_{j}^{i}-p^{i})$, where *d*_0_ denotes the initial demand in the live streaming session, $e_{j}^{i}$ represents the sales effort level of anchor *j* in model *i*, and $e_{j}^{i}>0$, $\lambda_{j}$ indicates the influence power of anchor *j*, ${\text{p}}^{\text{i}}$ is the product price in model *i*, and $t_{j}^{i}$ signifies the proportion of live streaming time allocated to anchor *j* in model *i*. We assume that for any chosen live streaming model, the sum of the time proportions allocated to all anchors equals one. This assumption allows us to standardize our analysis based on an average live streaming session duration. The demand function for the AI anchor is denoted by $d_{a}^{i}=t_{a}^{i}(d_{0}+\lambda_{a}s^{i}-p^{i})$, where ${\text{s}}^{\text{i}}$ represents the intelligence level of the AI anchor in model *i*.

In the context of regular anchor and KOL live streaming, anchors independently determine their level of sales effort, which significantly influences consumer purchase intentions [24]. By incorporating the influence power $\lambda_{j}$ and sales effort $e_{j}^{i}$ of different anchors, this directly impacts demand $\lambda_{j}e_{j}^{i}$. For instance, under identical levels of sales effort, anchors with greater influence can typically attract and generate higher consumer demand. In contrast, the intelligence level of the AI anchor, which reflects its responsiveness, is influenced by the level of investment in AI technology [33]. A higher intelligence level allows the AI anchor to respond more swiftly and accurately to customer inquiries and interactions, thus narrowing the performance gap between AI and human anchors [45]. The demand for the AI anchor is determined by both its influence power $\lambda_{a}$ and intelligence level ${\text{s}}^{\text{i}}$, which collectively translate into demand $\lambda_{a}s^{i}$. Importantly, when sales effort or intelligence levels are comparable, KOLs typically generate the highest demand.

Both the regular anchor and the KOL incur costs in their marketing efforts. Drawing on previous literature [36, 37, 39, 41], we assume that the cost of anchor’s efforts is quadratically related to their effort levels, denoted as $n_{j}{\left(e_{j}^{i}\right)}^{2}/2\;$, *n*_*j*_ represents the marketing effort cost coefficient for anchor *j*. AI anchors are typically either self-funded by brands or developed and provided by live streaming platforms for brand use. For instance, well-known brands such as Philips and L’Oréal have invested in and utilized AI anchors for live commerce, while platforms like ByteDance’s Douyin have developed AI anchors for merchants on their platform. It has been observed that as AI anchors become more intelligent and professional, their associated investment costs increase. Thus, referring to the study by Niu *et al*. [27], we assume a quadratic relationship between the investment cost and intelligence level of the AI anchor, represented as $m{\left(s^{i}\right)}^{2}/2\;$, represents the investment cost coefficient for the AI anchor.

Furthermore, we observe variation in commission rates among different types of anchors for their sales shares during live streaming sessions [21]. Regular anchors and KOLs earn commissions based on their sales within these sessions, with respective rates denoted as $\alpha_{r}$ and $\alpha_{k}$,. Reflecting industry norms, we assume that the commission rate for regular anchors is lower than that for KOLs, represented as $\alpha_{r}<\alpha_{k}$, $0<\alpha_{r}\leq 1/4\;$and $0<\alpha_{k}\leq 1/2\;$ [5]. In practice, the brand does not incur commission costs for the AI anchor, irrespective of the investor. If the live streaming platform funds the AI anchor, the brand is only required to pay a nominal usage fee. Given its minimal impact, we assume this usage fee for the AI anchor to be zero.

In addition to the aforementioned expenses, the brand is obligated to pay a commission to the live streaming platform as a usage fee for each event [24]. We assume that this commission rate is consistent with actual platform commission rates. For example, commission rates on Tmall Live typically range from 10% to 20%, whereas on Amazon.com, they vary from 6% to 25% [14, 46, 47]. Consequently, we hypothesize a commission rate that aligns with a range $0<\alpha_{p}\leq 1/3\;$. Additionally, the brand pays a fixed pit fee *F* to the KOL. Considering that the base salary of the regular anchor in this study is significantly lower than their commission rates, we assume the base salary for the regular anchor to be zero. We summarized all notations of the paper in Table 4.

**Table 4:** Summary of basic notation.

Variables	Description
$e_{j}^{i}$	The sales effort level of anchor *j* in Model *i*
${\text{s}}^{\text{i}}$	The intelligence level of AI anchor in Model *i*
${\text{p}}^{\text{i}}$	The product price in Model *i*
Parameters	Description
*d*_0_	The initial demand in the live streaming room
$t_{j}^{i}$	The proportion of live streaming time allocated to anchor *j* in Model *i*
$\lambda_{j}$	The influence power of anchor *j*
*n*_*j*_	The sales effort cost coefficient for anchor *j*
*m*	The investment cost coefficient for AI anchor
*F*	A fixed pit fee
$\alpha_{j}$	The commission rate of anchor *j*
$d_{j}^{i}$	The market demand generated by anchor *j* in Model *i*
$\pi_{j}^{i}$	The profit of entity *j* in Model *i*

In S1 Appendix A, we illustrate the event sequences for the discussed six models. For instance, in Model PN: the process initiates with the brand establishing the retail price in the initial stage. Subsequently, the live streaming platform sets the intelligence level of the AI anchor in the second stage. In the final stage, the regular anchor decides on their sales effort level. In Model PK: the sequence commences with the KOL determining the retail price and their effort level in the first stage. The intelligence level of the AI anchor is then set by the live streaming platform in the second stage. Lastly, the regular anchor selects their sales effort level in the third stage.

## 4 Scenario without KOL involvement

This section explores the choice of live streaming models that do not involve KOLs. Given that neither the regular anchor nor the AI anchor possesses the authority to set prices [27], pricing decisions for products are made by the brand. Using Model NN as our benchmark, we introduce an AI anchor financed either by the brand or the live streaming platform to assess its impact on live streaming. Our analysis focuses on determining the optimal decisions and maximizing profits for the participants in the supply chain across three configurations: the regular anchor model, the combined regular and AI anchor model with the brand covering investment costs, and the combined model where the live streaming platform covers investment costs.

### 4.1 Model analysis

In Model NN, which solely features the regular anchor for live streaming, the proportion of live streaming time attributed to the regular anchor is $t_{r}^{NN}=1$. With the introduction of the AI anchor (i.e., Model BN and PN), the proportion of live streaming time allocated to the regular anchor shifts to $t_{r}^{BN}=t_{r}^{PN}=\delta$, while that of AI anchor is represented by $t_{a}^{BN}=t_{a}^{PN}=1-\delta$, δ∈(0,1). Meanwhile, to ensure that the decision-making and profits of supply chain members in various live streaming modes are positive, we impose the following conditions: $n_{r}-\alpha_{r}{\lambda_{r}}^{2}>0$, $m-(1-\delta )\alpha_{p}{\lambda_{a}}^{2}>0$. In Model NN, the profits for the regular anchor and the brand are as follows:

$$\pi_{r}^{NN}=\alpha_{r}d_{r}^{NN}p^{NN}-n_{r}{\left(e_{r}^{NN}\right)}^{2}/2\;$$ (1)

$$\pi_{b}^{NN}=(1-\alpha_{r}-\alpha_{p})d_{r}^{NN}p^{NN}$$ (2)

In Model BN, where the brand funds an AI anchor for live streaming, both the regular anchor and the AI anchor are active on live streaming platforms. While deploying an AI anchor enables the brand to avoid sharing sales revenue, it also requires the brand to absorb the investment costs associated with the AI anchor. The resulting profit for the regular anchor and the brand are as follows:

$$\pi_{r}^{BN}=\alpha_{r}d_{r}^{BN}p^{BN}-n_{r}{\left(e_{r}^{BN}\right)}^{2}/2\;$$ (3)

$$\pi_{b}^{BN}=(1-\alpha_{r}-\alpha_{p})d_{r}^{BN}p^{BN}+(1-\alpha_{p})d_{a}^{BN}p^{BN}-m{\left(s^{BN}\right)}^{2}/2\;$$ (4)

In Model PN, the brand opts to use an AI anchor financed by the live streaming platform. This arrangement means the brand forfeits control over the AI anchor’s intelligence settings to the platform, but does not bear the AI anchor’s investment costs. The profit outcomes for the regular anchor, the live streaming platform, and the brand are delineated as follows:

$$\pi_{r}^{PN}=\alpha_{r}d_{r}^{PN}p^{PN}-n_{r}{\left(e_{r}^{PN}\right)}^{2}/2\;$$ (5)

$$\pi_{p}^{PN}=\alpha_{p}(d_{r}^{PN}+d_{k}^{PN}+d_{a}^{PN})p^{PN}-m{\left(s^{PN}\right)}^{2}/2\;$$ (6)

$$\pi_{b}^{PN}=(1-\alpha_{r}-\alpha_{p})d_{r}^{PN}p^{PN}+(1-\alpha_{p})d_{a}^{PN}p^{PN}$$ (7)

We solved the profit maximization problem using backward induction, and the equilibrium results are presented in Table 5 of S1 Appendix A.

### 4.2 Impact of AI anchors

In this section, we compare the decisions and profits of supply chain members in scenarios without KOL involvement. To better investigate the impacts of $\lambda_{r}$, $\lambda_{a}$ and δ on the decisions and profits of supply chain members, we follow the methodologies established by Niu *et al*. [27] and Chen [48]. We assume *m* = *n*_*r*_ = 1.

**Proposition 1.** *In scenarios without KOL involvement,*

*(1) if $0<\lambda_{r}<\lambda_{r1}$, then $\pi_{b}^{NN}<\pi_{b}^{BN}$;*

*(2) if $0<\lambda_{r}<\lambda_{r2}$, then $\pi_{b}^{NN}<\pi_{b}^{PN}$.*

Proposition 1 highlights that when the influence of the regular anchor is minimal (i.e., $0<\lambda_{r}<\lambda_{r1}$), the brand will explore alternatives to invigorate store activity. Under such circumstances, integrating an AI anchor can address the shortcomings of the regular anchor and enhance the overall sales performance of the live streaming store. Conversely, if the regular anchor is highly influential (i.e., $\lambda_{r1}<\lambda_{r}$), the introduction of an AI anchor may be unnecessary and could potentially hinder operations.

**Corollary 1.** *In Model BN and PN, the sensitivity analysis of parameters $\lambda_{r}$ and $\lambda_{a}$ is as follows:*

*(1)$\frac{\partial e_{r}^{BN}}{\partial \lambda_{r}}>0$, $\frac{\partial s^{BN}}{\partial \lambda_{r}}>0$, $\frac{\partial p^{BN}}{\partial \lambda_{r}}>0$, $\frac{\partial \pi_{b}^{BN}}{\partial \lambda_{r}}>0$, $\frac{\partial e_{r}^{BN}}{\partial \lambda_{a}}>0$, $\frac{\partial s^{BN}}{\partial \lambda_{a}}>0$, $\frac{\partial p^{BN}}{\partial \lambda_{a}}>0$, $\frac{\partial \pi_{b}^{BN}}{\partial \lambda_{a}}>0$.*

*(2)$\frac{\partial e_{r}^{PN}}{\partial \lambda_{r}}>0$, $\frac{\partial s^{PN}}{\partial \lambda_{r}}>0$, $\frac{\partial p^{PN}}{\partial \lambda_{r}}>0$, $\frac{\partial \pi_{b}^{PN}}{\partial \lambda_{r}}>0$, $\frac{\partial e_{r}^{PN}}{\partial \lambda_{a}}>0$, $\frac{\partial s^{PN}}{\partial \lambda_{a}}>0$, $\frac{\partial p^{PN}}{\partial \lambda_{a}}>0$, $\frac{\partial \pi_{b}^{PN}}{\partial \lambda_{a}}>0$.*

Corollary 1 suggests that the deployment of an AI anchor results in enhanced intelligence levels from the AI anchor and increased sales efforts levels from the regular anchor, irrespective of whether it is the regular anchor’s or the AI anchor’s influence that is expanding. When the regular anchor’s influence increases, they will naturally intensify their sales effort. In response, to drive sales and ensure the AI anchor effectively complements the regular anchor, the brand or live streaming platform may increase investment in the AI anchor to enhance its intelligence level. Conversely, if the AI anchor’s influence intensifies, the brand or live streaming platform will capitalize on this by further enhancing the AI anchor’s intelligence to amplify sales. Concurrently, the regular anchor, motivated by higher product prices, will intensify efforts to capture a larger portion of the sales revenue. From a management perspective, if the brand finances the AI anchor, the increased investment costs are likely to lead to higher product prices to recoup this expenditure, thereby elevating profits. Alternatively, if the live streaming platform funds the AI anchor, the simultaneous increase in the AI anchor’s intelligence and the regular anchor’s marketing efforts will prompt the brand to raise product prices to boost its profits. This highlights a strategic consideration for brands: employing a high-influence regular or AI anchor can enhance profitability but may also result in elevated product prices, potentially eroding the price competitiveness.

**Corollary 2.** *In Model BN and PN, the sensitivity analysis of parameter δ is as follows:*

*(1)$\frac{\partial e_{r}^{BN}}{\partial \delta }>0$,$\frac{\partial e_{r}^{PN}}{\partial \delta }>0$,$\frac{\partial s^{BN}}{\partial \delta }<0$,$\frac{\partial s^{PN}}{\partial \delta }<0$.*

*(2) $\frac{\partial p^{BN}}{\partial \delta }<0$ if $0<\lambda_{r}<\lambda_{r3}$; $\frac{\partial p^{RN}}{\partial \delta }<0$ if $0<\lambda_{r}<\lambda_{r4}$.*

*(3) $\frac{\partial \pi_{b}^{BN}}{\partial \delta }<0$ if $0<\lambda_{r}<\lambda_{r5}$; $\frac{\partial \pi_{b}^{PN}}{\partial \delta }<0$.*

As the regular anchor’s live streaming time proportion increases, it signals the brand’s increased trust and expectations for that anchor. Corollary 2(1) indicates that without KOL participation, when a brand decides to use AI anchors—regardless of who funds them—increasing the share of live streaming time allocated to regular anchors incentivizes them to exert greater effort but reduces the intelligence level of AI anchors. This can be intuitively understood: when the share of live streaming time for regular anchors increases after AI anchors are introduced, it signals the brand’s higher valuation of regular anchors. Consequently, regular anchors feel more motivated to exert effort. Meanwhile, this shift reduces the investment in AI anchors by both brands and live streaming platforms, resulting in a decrease in AI anchors’ intelligence levels. Corollary 2(2) points out that if the regular anchor’s influence is weak (i.e., $0<\lambda_{r}<\lambda_{r3}$ or $0<\lambda_{r}<\lambda_{r4}$), the brand may reduce product prices as the regular anchor’s live streaming time increases, supporting the regular anchor and attempting to drive demand through price modifications. Corollary 2(3) discusses the differing outcomes depending on who finances the AI anchor. If the brand funds the AI anchor, only a low influence from the regular anchor (i.e., $0<\lambda_{r}<\lambda_{r5}$) paired with increased live streaming time will reduce profits, since the brand can effectively manage the AI anchor. However, if the live streaming platform funds the AI anchor, any increase in the regular anchor’s live streaming time generally leads to profit declines due to the brand’s lack of control and obligation to pay commissions. These insights emphasize the need for brands to strategically decide who should finance the AI anchor. Effective control over the AI anchor can lead to better integration and potentially higher returns, highlighting that absorbing the investment cost can be beneficial. This strategic decision is critical as different financing options can significantly impact outcomes.

**Proposition 2.** *(1) If $\lambda_{a}>\lambda_{a1}$, then ${\text{p}}^{\text{NN}}<{\text{p}}^{\text{BN}}$; otherwise, ${\text{p}}^{\text{NN}}>{\text{p}}^{\text{BN}}$.*

*(2) If $\lambda_{a}>\lambda_{a2}$, then ${\text{p}}^{\text{NN}}<{\text{p}}^{\text{PN}}$; otherwise, ${\text{p}}^{\text{NN}}>{\text{p}}^{\text{PN}}$.*

*(3) ${\text{p}}^{\text{BN}}>{\text{p}}^{\text{PN}}$.*

**Fig 3:**
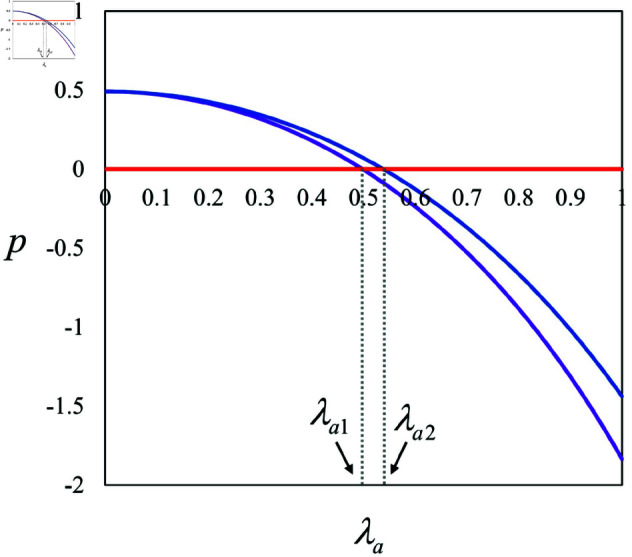
Illustration of Proposition 2(1) and (2).

Proposition 2 indicates that in scenarios without KOL participation, regardless of the investor, a high-influence AI anchor (i.e., $\lambda_{a}>\lambda_{a1}$ or $\lambda_{a}>\lambda_{a2}$) leads the brand to set higher product prices, as illustrated in Fig 3. This adjustment is primarily because the brand recognizes the AI anchor’s significant influence and aims to capitalize on it by boosting sales through increased pricing. Additionally, it is observed that an AI anchor financed by the brand tends to result in a higher product price compared to one funded by the live streaming platform. This difference arises because the brand, having invested in the AI anchor, needs to recoup the investment costs, prompting it to set a higher product price. Conversely, when the AI anchor is funded by the live streaming platform, the brand incurs no direct investment costs, allowing it to offer a lower price.

**Proposition 3.** $e_{r}^{NN}>e_{r}^{BN}>e_{r}^{PN}$. 

Proposition 3 reveals that without KOL participation, the introduction of AI anchors—regardless of the investor—reduces the effort level of regular anchors. This finding highlights that for regular anchors, the presence of AI anchors does not incentivize greater effort but instead discourages it. Furthermore, regular anchors show less willingness to exert effort when AI anchors are funded by live streaming platforms compared to brand-funded AI anchors. Combined with Proposition 3(3), it becomes evident that when brands utilize AI anchors funded by platforms, they set lower product prices. Consequently, regular anchors may worry that their efforts will not yield sufficient rewards, leading to reduced effort in Model PN.

**Proposition 4.** In scenarios without KOL involvement, there is ${\text{s}}^{\text{BN}}>{\text{s}}^{\text{PN}}$.

Proposition 4 suggests that in settings without KOL participation, an AI anchor financed by the brand typically displays greater intelligence compared to one funded by the live streaming platform. This observation is consistent with practical scenarios where brands, aiming to enhance market competitiveness and optimize their live streaming operations, are more likely to allocate substantial resources towards the development and refinement of the AI anchor. In contrast, live streaming platforms, which primarily function as broadcast channels, may view the AI anchor as just additional function, resulting in a potentially lower level of intelligence development for such AI anchors.

**Proposition 5.** In scenarios without KOL involvement, there is $\pi_{b}^{BN}>\pi_{b}^{PN}$.

Integrating insights from Proposition 5, it becomes clear that with a regular anchor of moderate influence (i.e., $\lambda_{r2}<\lambda_{r}<\lambda_{r1}$), the brand benefits from investing in its own AI anchor to facilitate better coordination between the two anchors, thereby boosting profits. When the regular anchor’s influence is minimal (i.e., $0<\lambda_{r}<\lambda_{r2}$), utilizing an AI anchor financed by the live streaming platform proves more advantageous for live streaming operations than not using an AI anchor at all, leading to increased profits. Furthermore, Proposition 5 shows that even with a low-influence regular anchor, the brand often opts to assume the extra investment costs to deploy its own AI anchor, rather than depending on the platform’s AI. Drawing from Propositions 3 and 4, it is evident that an AI anchor funded by the brand more effectively enhances the live stream, stimulates the regular anchor, and improves intelligence, culminating in greater profits for the brand. This rationale supports why JD Cloud’s Yanxi team developed AI anchors have been successfully implemented in JD Home Appliances & Furniture and JD Supermarket’s live streaming sessions, achieving optimal streaming outcomes.

To understand when the brand’s choice of mode can lead to a “win-win” situation, where all members of the supply chain see maximized profits, we first examine scenarios without an AI anchor. For a clearer comprehension, we illustrate the “win-win” region through numerical simulations with specified parameter settings: *d*_0_ = 20, $\lambda_{r}=0.5$, $\alpha_{r}=0.2$, $\alpha_{p}=0.3$, $\lambda_{a}\in (0,1)$.

**Fig 4:**
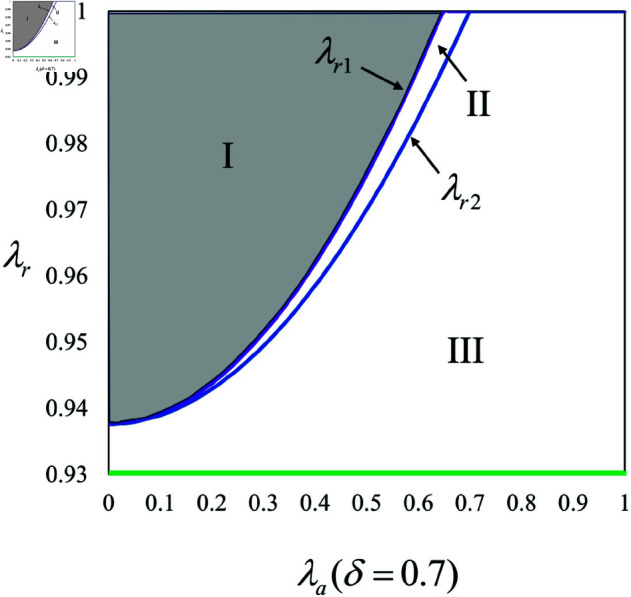
Win-win situation for the Regular anchor and the Brand without KOL involvement.

In scenarios without KOL involvement, a “win-win” situation emerges predominantly when the regular anchor’s live streaming duration is substantial and the regular anchor’s influence is elevated, as region I of Fig 4 show. Integrating insights from Proposition 1 with Fig 4, it is evident that when the regular anchor’s live streaming duration is extensive and their influence substantial, the brand opts out of deploying an AI anchor, thereby maximizing the regular anchor’s profits. Conversely, when the regular anchor’s live streaming duration is limited or their influence is minimal, the brand considers introducing an AI anchor to address these deficiencies, as depicted in regions II and III of Fig 4. This strategy, although potentially detrimental to the regular anchor’s interests (i.e., $\pi_{r}^{NN}>\pi_{r}^{BN}$ or $\pi_{r}^{NN}>\pi_{r}^{PN}$) and posing a risk of replacement, is often necessary from the brand’s perspective. It also follows from Fig 4 that even though employing an AI anchor might affect the regular anchor’s interests adversely, the ultimate decision on the live streaming mode rests with the brand. If the brand opts for an AI anchor, it is crucial to assess whether the live streaming mode can sustain stability under such conditions. According to Proposition 5 and Fig 4, in the absence of KOL involvement, if the brand decides to employ an AI anchor, a preference for the brand to handle the investment emerges, fostering a “win-win” scenario. This preference indicates that if the decision to use an AI anchor is made, the brand will likely invest in it directly. As Fig 4 illustrates, the regular anchor also favors an AI anchor funded by the brand, that is, $\pi_{r}^{BN}>\pi_{r}^{PN}$ . Therefore, whenever the brand employs an AI anchor, its choice to self-invest aligns with the regular anchor’s preference, creating a stable operational mode.

**Observation 1.** *In scenarios without KOL involvement,*

*(1) increasing the regular anchor’s proportion of live streaming time can augment the potential for mutual benefits;*

*(2) the integration of an AI anchor can notably enhance the stability of the live streaming mode.*

As indicated by Fig 4, a rise in the regular anchor’s live streaming time suggests a greater dependence on the regular anchor’s performance, which aligns the decision-making processes of both the brand and the regular anchor, thereby mitigating potential disagreements and uncertainties. This alignment may foster a more stable environment conducive to pursuing higher profits. Furthermore, while the introduction of an AI anchor might initially seem to undermine the regular anchor’s interests, it actually synchronizes the preferences of the regular anchor and the brand. This synchronization reduces the unpredictability of live streaming outcomes, thus contributing to the stability of the live streaming mode. These insights underscore that when choosing a live streaming mode, the brand must thoroughly consider the role of the regular anchor to balance the interests within the supply chain. Additionally, to bolster the stability of store live streaming, an AI anchor could be a strategic addition, as it diminishes uncertainties and promotes more consistent choices in live streaming mode.

## 5 Scenario with KOL involvement

In scenarios involving KOL participation during live streaming, there is a notable shift in the dynamics of pricing decisions and sequences. KOLs, with their extensive fan bases and consistent conversion rates, typically command pricing authority [19], compelling other anchors to adopt a pricing strategy that follows the KOL’s lead. This section explores the selection of live streaming modes that include a KOL (denoted with the subscript ‘k’), using a hybrid model of regular anchors and KOLs as the foundational framework. Building on the NK model, we also examine the effects of incorporating an AI anchor, funded either by the brand or the live streaming platform, on the dynamics of live streaming.

### 5.1 Model analysis

Since Model NK only involves regular and KOL anchors for live streaming, the live streaming time proportion for the regular anchor is $t_{r}^{NK}=\delta$, and that for the KOL is $t_{k}^{NK}=1-\delta$. Upon introducing the AI anchor (i.e., Model BK and PK), three different types of anchors coexist. At this point, the live streaming time proportion for the regular anchor is $t_{r}^{BK}=t_{r}^{PK}=\delta$, for the KOL is $t_{k}^{BK}=t_{k}^{PK}=\mu$, and for the AI anchor is $t_{a}^{BK}=t_{a}^{PK}=1-\delta -\mu$, μ∈(0,1). Meanwhile, to ensure that the decision-making and profits of supply chain members in various live streaming modes are positive, we impose the following conditions: $2n_{k}-(1-\delta )\alpha_{k}{\lambda_{k}}^{2}>0$, $2n_{k}-\mu \alpha_{k}{\lambda_{k}}^{2}>0$.

In the Model NK, the brand invites KOL to conduct live streaming, both the regular anchor and KOL coexist on live streaming platforms, and the brand bears a high percentage of sales commissions for the KOL. The profits for the regular anchor, the KOL, and the brand are as follows:

$$\pi_{r}^{NK}=\alpha_{r}d_{r}^{NK}p^{NK}-n_{r}{\left(e_{r}^{NK}\right)}^{2}/2\;$$ (8)

$$\pi_{k}^{NK}=\alpha_{k}d_{k}^{NK}p^{NK}-n_{k}{\left(e_{k}^{NK}\right)}^{2}/2\;+F$$ (9)

$$\pi_{b}^{NK}=(1-\alpha_{r}-\alpha_{p})d_{r}^{NK}p^{NK}+(1-\alpha_{k}-\alpha_{p})d_{k}^{NK}p^{NK}-F$$ (10)

In Model BK, the brand not only invites a KOL for live streaming but also invests in an AI anchor. This setup leads to the simultaneous involvement of a regular anchor, a KOL, and an AI anchor in live streaming activities on platforms. While the introduction of an AI anchor allows the brand to reduce a portion of the sales commissions, it also necessitates bearing the investment costs associated with the AI anchor. The profit distribution among the regular anchor, the KOL, and the brand are as follows:

$$\pi_{r}^{BK}=\alpha_{r}d_{r}^{BK}p^{BK}-n_{r}{\left(e_{r}^{BK}\right)}^{2}/2\;$$ (11)

$$\pi_{k}^{BK}=\alpha_{k}d_{k}^{BK}p^{BK}-n_{k}{\left(e_{k}^{BK}\right)}^{2}/2\;+F$$ (12)

$$\pi_{b}^{BK}=(1-\alpha_{r}-\alpha_{p})d_{r}^{BK}p^{BK}+(1-\alpha_{k}-\alpha_{p})d_{k}^{BK}p^{BK}\;\;+(1-\alpha_{p})d_{a}^{BK}p^{BK}-\frac{m(s^{BK}{)}^{2}}{2}-F,$$ (13)

In Model PK, the brand engages a KOL and uses an AI anchor funded by the live streaming platform for the live streaming. This arrangement differs from the BK model in that the live streaming platform bears the investment costs of the AI anchor, thereby gaining control over the AI anchor’s level of intelligence. The profit outcomes for the regular anchor, the KOL, the live streaming platform, and the brand are as follows:

$$\pi_{r}^{PK}=\alpha_{r}d_{r}^{PK}p^{PK}-n_{r}{\left(e_{r}^{PK}\right)}^{2}/2\;$$ (14)

$$\pi_{k}^{PK}=\alpha_{k}d_{k}^{PK}p^{PK}-n_{k}{\left(e_{k}^{PK}\right)}^{2}/2\;+F$$ (15)

$$\pi_{p}^{PK}=\alpha_{p}(d_{r}^{PK}+d_{k}^{PK}+d_{a}^{PK})p^{PK}-m{\left(s^{PK}\right)}^{2}/2\;$$ (16)

$$\pi_{b}^{PK}=(1-\alpha_{r}-\alpha_{p})d_{r}^{PK}p^{PK}+(1-\alpha_{k}-\alpha_{p})d_{k}^{PK}p^{PK}+(1-\alpha_{p})d_{a}^{PK}p^{PK}-F$$ (17)

We addressed the profit maximization issue through backward induction, and the equilibrium outcomes are detailed in Table 6 of S1 Appendix A.

### 5.2 Impact of AI anchors

In this section, we explore the decision-making processes and profit outcomes for supply chain members when a KOL is involved. This paper specifically focuses on the variables $\lambda_{r}$, $\lambda_{k}$, $\lambda_{a}$, and δ, assuming $m=n_{r}=n_{k}=1$. To understand the impact of deploying an AI anchor in scenarios with a KOL, we initially compare the brand’s profitability across the NK, BK, and PK models to determine the conditions under which the brand might opt to use an AI anchor. Given the complexity of direct calculations, we employ numerical simulations to more effectively analyze and illustrate how profits vary across different live streaming models. We also highlight how critical model parameters influence the brand’s profit outcomes. The parameters used in our simulations are set as follows: $\alpha_{k}=0.5$, $\lambda_{k}\in (0,2)$.

**Fig 5:**
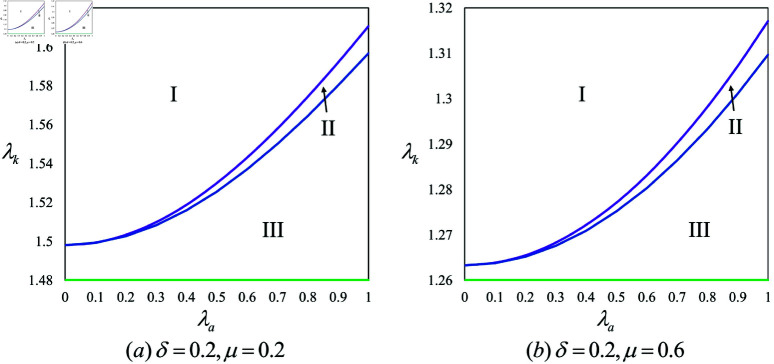
Comparison of the Brand’s profits with KOL involvement.

In scenarios with a KOL, the decision to use an AI anchor hinges on the regular anchor’s live streaming time and the KOL’s influence. Specifically, if the regular anchor’s live streaming time is minimal and the KOL’s influence is moderate (If δ is less and $\lambda_{k}$ is not high), or if the regular anchor’s live streaming time is significant (If δ is high), the brand will likely deploy an AI anchor (i.e., $\pi_{b}^{NK}<\pi_{b}^{BK}$ or $\pi_{b}^{NK}<\pi_{b}^{PK}$), as depicted in regions II and III of Fig 5a and 5b. The brand’s profits are significantly shaped by the KOL’s influence and the proportion of time the regular anchor streams. When the regular anchor’s streaming time is low and the KOL’s influence is substantial, the brand typically refrains from employing an AI anchor, regardless of the KOL’s streaming duration, as region I of Fig 5a and 5b show. This decision stems from the minimal impact of the regular anchor under these conditions, and the dominant presence of a strong KOL, under which neither the regular anchor nor the AI anchor are likely to contribute significantly. Despite potential savings on commission costs, the use of an AI anchor is deemed unnecessary. On the other hand, when the regular anchor’s live streaming time is extensive, suggesting a relatively minor role for the KOL, the brand tends to adopt an AI anchor to sidestep the regular anchor’s commissions, even at the cost of additional investment. This approach highlights the need for brands to carefully evaluate the influence and streaming durations of different anchors, ensuring a strategic mix that leverages the unique strengths of each to enhance the overall effectiveness of live streaming.

**Observation 2.** *In scenarios with KOL Involvement, a greater allocation of live streaming time to the KOL decreases the probability of the brand deploying an AI anchor. Conversely, an increase in the live streaming time allocated to the regular anchor may encourage the brand to consider the use of an AI anchor.*

Moreover, upon comparing Fig 5a and 5b, we observe that enhancing the KOL’s live streaming time allows the KOL to more effectively leverage their influence, convincing the brand that the combined efforts of the KOL and the regular anchor are sufficient without the integration of an AI anchor. Conversely, if the regular anchor’s live streaming time is increased, allowing them to demonstrate improved performance, the comparative advantage of the KOL diminishes, thus bolstering the brand’s inclination to employ an AI anchor.

**Corollary 3.** *In Model BK and PK, the sensitivity analysis of parameters $\lambda_{r}$, $\lambda_{k}$ and $\lambda_{a}$ is as follows:*

*(1) $\frac{\partial e_{r}^{BK}}{\partial \lambda_{r}}>0$, $\frac{\partial \pi_{b}^{BK}}{\partial \lambda_{r}}>0$, $\frac{\partial s^{BK}}{\partial \lambda_{a}}>0$, $\frac{\partial \pi_{b}^{BK}}{\partial \lambda_{a}}>0$, $\frac{\partial e_{r}^{BK}}{\partial \lambda_{k}}>0$, $\frac{\partial e_{k}^{BK}}{\partial \lambda_{k}}>0$, $\frac{\partial s^{BK}}{\partial \lambda_{k}}>0$, $\frac{\partial p^{BK}}{\partial \lambda_{k}}>0$.*

*(2) $\frac{\partial e_{r}^{PK}}{\partial \lambda_{r}}>0$, $\frac{\partial \pi_{b}^{PK}}{\partial \lambda_{r}}>0$, $\frac{\partial s^{PK}}{\partial \lambda_{a}}>0$, $\frac{\partial \pi_{b}^{PK}}{\partial \lambda_{a}}>0$, $\frac{\partial e_{r}^{PK}}{\partial \lambda_{k}}>0$, $\frac{\partial e_{k}^{PK}}{\partial \lambda_{k}}>0$, $\frac{\partial s^{PK}}{\partial \lambda_{k}}>0$, $\frac{\partial p^{PK}}{\partial \lambda_{k}}>0$.*

*(3) If $0<\lambda_{k}<\lambda_{k1}$, then $\frac{\partial \pi_{b}^{BK}}{\partial \lambda_{k}}>0$; if $0<\lambda_{k}<\lambda_{k2}$, then $\frac{\partial \pi_{b}^{PK}}{\partial \lambda_{k}}>0$.*

**Fig 6:**
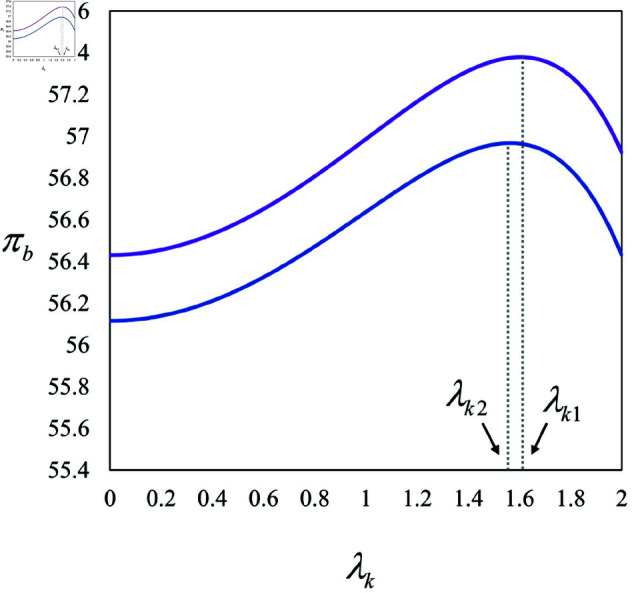
Illustration of Corollary 3(3).

According to Corollary 3, in both the BK and PK models, we note the following impacts of varying the influence levels of different anchors and KOLs: (a) An increase in the influence allotted to either the regular anchor or the KOL encourages the regular anchor to increase their effort, a trend consistent across both models. (b) Elevating the influence of either the KOL or the AI anchor encourages investors to boost the AI anchor’s intelligence. (c) A higher influence assigned to the KOL is likely to drive up product prices. (d) Boosting the influence of the regular anchor or the AI anchor, or increasing the KOL’s influence within a specified lower threshold (i.e., $0<\lambda_{k}<\lambda_{k1}$ or $0<\lambda_{k}<\lambda_{k2}$, as illustrated in Fig 6), can significantly enhance the brand’s profits. It is clear that boosting the regular anchor’s influence motivates them to exert more effort. Similarly, enhancing the AI anchor’s influence prompts investors to upgrade its intelligence capabilities. Moreover, without impacting product prices, increased efforts from the regular anchor or advancements in the AI anchor’s intelligence can substantially improve the brand’s profitability. During live streams that feature a KOL, selecting one with greater influence generally results in increased effort from the KOL, though this also incurs higher costs. To offset these costs, the KOL may raise product prices, thereby augmenting their own profits. This price increase motivates investors of both the regular and AI anchors to seek higher profits, leading them to intensify efforts and improve the AI anchor’s intelligence.

**Corollary 4.** *In Model BK and PK, the sensitivity analysis of parameters δ and μ is as follows:*

*(1) $\frac{\partial e_{r}^{BK}}{\partial \delta }>0$, $\frac{\partial s^{BK}}{\partial \delta }<0$, $\frac{\partial e_{r}^{BK}}{\partial \mu }>0$, $\frac{\partial e_{k}^{BK}}{\partial \mu }>0$, $\frac{\partial p^{BK}}{\partial \mu }>0$.*

*(2) $\frac{\partial e_{r}^{PK}}{\partial \delta }>0$, $\frac{\partial s^{PK}}{\partial \delta }<0$, $\frac{\partial e_{r}^{PK}}{\partial \mu }>0$, $\frac{\partial e_{k}^{PK}}{\partial \mu }>0$, $\frac{\partial p^{PK}}{\partial \mu }>0$.*

*(3) $\frac{\partial s^{BK}}{\partial \mu }<0$ and $\frac{\partial s^{PK}}{\partial \mu }<0$ if $0<\lambda_{k}<\sqrt{\frac{2}{(1-\delta )\alpha_{k}}}$.*

*(4) $\frac{\partial \pi_{b}^{BK}}{\partial \delta }<0$ if $0<\lambda_{r}<\lambda_{r6}$, $0<\lambda_{k}<\lambda_{k3}$; $\frac{\partial \pi_{b}^{PK}}{\partial \delta }<0$ if $0<\lambda_{r}<\lambda_{r7}$, $0<\lambda_{k}<\lambda_{k4}$.*

Corollary 4(1) and (2) reveal that in settings involving a KOL, the decision by a brand to increase the regular anchor’s share of live streaming time, in either the BK or PK model, signals a higher valuation of the regular anchor. Recognizing this appreciation, the regular anchor is likely to increase their effort. In contrast, investors have similar perceptions of the AI anchor’s contribution, leading them generally to decrease their investments in its intelligence capabilities when its live streaming share is reduced. Conversely, allocating more live streaming time to the KOL not only spurs greater effort from them to boost demand but also leads to higher operational costs. To cover these costs, the KOL may raise product prices, which, in turn, prompts the regular anchor to intensify their efforts as well. Corollary 4(3) suggests that in cases where a KOL is involved and the brand opts to use an AI anchor, a low influence of the KOL (i.e., $0<\lambda_{k}<\sqrt{\frac{2}{(1-\delta )\alpha_{k}}}$) indicates a less than optimal overall live streaming performance. Increasing the KOL’s live streaming share under these conditions may convince investors that the AI anchor’s return on investment is low, leading to decreased funding for its intelligence. Lastly, Corollary 4(4) points out that in scenarios with KOL involvement, whether in the Model BK or PK, only when both the regular anchor and the KOL have low influence (i.e., $0<\lambda_{r}<\lambda_{r6}$, $0<\lambda_{k}<\lambda_{k3}$ or $0<\lambda_{r}<\lambda_{r7}$, $0<\lambda_{k}<\lambda_{k4}$) does increasing the regular anchor’s live streaming share result in reduced profits for the brand. This is likely because when both figures wield low influence, it reflects poorly on the live streaming effectiveness, and increasing the less influential anchor’s screen time only aggravates the issue, further harming the brand’s profitability.

**Proposition 6.** *(1) $p^{NK}>p^{BK}=p^{PK}$, $e_{r}^{NK}>e_{r}^{BK}=e_{r}^{PK}$, $e_{k}^{NK}>e_{k}^{BK}=e_{k}^{PK}$.*

*(2) ${\text{s}}^{\text{BK}}>{\text{s}}^{\text{PK}}$.*

Proposition 1(1) indicates that introducing an AI anchor can lead to diminished efforts from both the regular anchor and the KOL, accompanied by a reduction in product prices. This introduction often means that the AI anchor either replaces or augments the roles of the regular anchor and KOL, making the brand less dependent on human anchors. This reduced dependency results in decreased exertion from both the regular anchor and the KOL compared to scenarios without an AI anchor. As the KOL sets the product prices, their decreased effort not only results in lower prices but also strategically, to enhance consumer demand, the KOL opts to set more attractive price points. Proposition 1(2) illustrates that AI anchors funded by brands exhibit higher intelligence levels than those funded by live streaming platforms. This discrepancy stems from the differing investment sources in scenarios where AI anchors are deployed. Despite similar conditions for other supply chain members and the KOL’s predominant role, the investor’s identity predominantly influences the AI anchor’s intelligence level. Brands tend to invest in AI anchors with the goal of maximizing store profits, leading to higher intelligence investments. Conversely, live streaming platforms, which bear the costs and only gain from a portion of sales, tend to invest more conservatively in AI intelligence, resulting in a moderately lower intelligence level for their AI anchors.

**Observation 3.** *In scenarios with KOL involvement, the decision by a brand to deploy an AI anchor does not influence the regular anchor or the KOL, regardless of whether the AI anchor is financed by the brand or the platform.*

Proposition 6 illustrates that variations in the investor of the AI anchor primarily affect only the control over the AI anchor, while perceptions of the live stream and decision-making processes remain consistent. Consequently, changes in the investor do not influence the regular anchor or the dominant KOL.

**Proposition 7.** *In scenarios with KOL involvement,, there is $\pi_{b}^{BK}>\pi_{b}^{PK}$.*

Proposition 7 highlights that a brand’s decision to deploy its own AI anchor remains constant across different allocations of live streaming time and varying levels of influence among anchors. As illustrated in Fig 5, when the regular anchor’s share of live streaming is minimal and the KOL wields moderate influence, the brand opts to invest in its own AI anchor. This strategy is adopted because the regular anchor’s limited impact on the live stream and the KOL’s moderate influence necessitate the introduction of an AI anchor to optimize engagement. Although choosing to invest in a proprietary AI anchor incurs higher costs compared to utilizing a platform-provided AI anchor, it enables the brand to better orchestrate its live streaming strategy and enhance profitability. Further analysis reveals that the brand consistently prefers investing in its own AI anchor, regardless of whether the regular anchor’s live streaming time is scant or extensive, or the KOL’s influence is strong or weak. This consistent preference is elucidated by Proposition 6, which indicates that despite the higher initial investment, the superior intelligence of the brand’s AI anchor significantly boosts its ability to generate profits. This strategic investment in AI technology allows the brand to maintain a competitive edge and achieve greater market success.

**Proposition 8.** *In scenarios with KOL involvement, there are*

*(1) If $\lambda_{r8}<\lambda_{r}$, then $\pi_{r}^{NK}>\pi_{r}^{BK}$, $\pi_{r}^{NK}>\pi_{r}^{PK}$ .*

*(2) $\pi_{k}^{NK}>\pi_{k}^{BK}$, $\pi_{k}^{NK}>\pi_{k}^{PK}$.*

Proposition 8(1) demonstrates that in scenarios involving a KOL, the introduction of an AI anchor adversely affects a regular anchor with strong influence (i.e., $\lambda_{r8}<\lambda_{r}$), irrespective of the AI anchor’s performance metrics. This outcome is understandable given that a highly influential regular anchor typically synergizes effectively with the KOL. Introducing an AI anchor, however, may disrupt this dynamic, potentially destabilizing the live stream environment and negatively impacting the regular anchor’s profitability. Conversely, if the regular anchor’s influence is minimal, the addition of an AI anchor effectively introduces a fresh dynamic that can enhance the overall live stream performance. Proposition 8(2) reveals that the introduction of an AI anchor, whether funded by the brand or the platform, impacts the KOL. The AI anchor offers consumers an alternative interaction point within the live stream. Despite the KOL’s dominant role, the AI anchor’s presence can attract a portion of the KOL’s viewership, consequently diminishing the KOL’s profitability. This indicates that the AI anchor poses a competitive threat to the KOL, altering the audience distribution and potentially the revenue streams in live stream settings.

**Fig 7:**
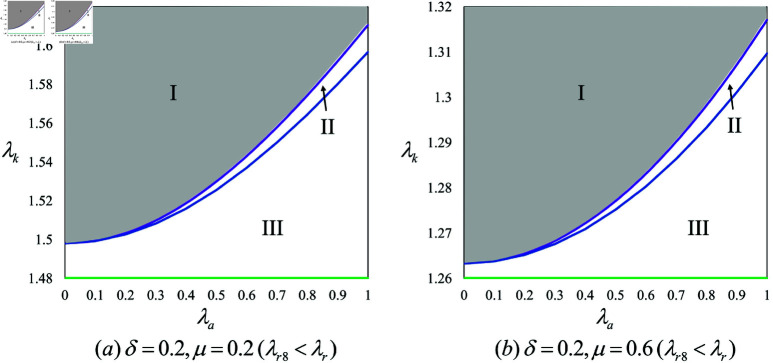
Triple-win situation for the Regular anchor, KOL and the Brand.

To explore the conditions under which the brand’s choice of model results in a “triple-win” scenario where the profits of all supply chain members are maximized we first examine situations without an AI anchor. Utilizing numerical simulations in conjunction with Fig 7 and Proposition 8, we clearly delineate the triple-win regions in scenarios that do not involve AI anchors.

As depicted in region I of Fig 7a and 7b, in scenarios involving a KOL, a “triple-win" outcome is achievable without the use of an AI anchor only under specific conditions: the regular anchor’s live streaming time is relatively brief, their influence is substantial, and the KOL’s influence is significant (i.e., if δ is less , $\lambda_{r10}<\lambda_{r}$ and $\lambda_{k}$ is high). Proposition 7 indicates that employing an AI anchor adversely affects the KOL. If the regular anchor also possesses considerable influence, they too may resist the introduction of an AI anchor. Additionally, Fig 7 demonstrates that when the regular anchor’s live streaming time is constrained and the KOL’s influence is strong, the brand is likely to decide against introducing an AI anchor. In such cases, opting not to deploy an AI anchor is the optimal strategy for maximizing the profits of all members of the supply chain, as evidenced in Fig 7. While the introduction of an AI anchor might undermine the interests of the KOL and potentially affect the regular anchor’s profits, it remains a profitable strategy for the brand. Given that the brand holds the decision-making authority over the live streaming model, the stability of the model must be assessed if the brand opts to introduce an AI anchor, ensuring that it can sustainably support the brand’s objectives.

**Proposition 9.** *In scenarios with KOL involvement, there are $\pi_{r}^{BK}=\pi_{r}^{PK}$, $\pi_{k}^{BK}=\pi_{k}^{PK}$ and $\pi_{b}^{BK}>\pi_{b}^{PK}$.*

When a brand opts to invest in its own AI anchor, this decision positively impacts all members of the supply chain, leading to a “triple-win" scenario. According to Proposition 8, regardless of the investor, the introduction of an AI anchor tends to result in losses for the KOL, and under certain conditions (i.e., $\lambda_{r10}<\lambda_{r}$), it may also adversely affect the regular anchor’s profits. However, changes in the investor of the AI anchor do not influence the decisions or profitability of the regular anchor or the KOL, but they significantly impact the brand. Proposition 7 further supports that a brand’s investment in its own AI anchor yields higher profits than if relying on a platform-provided AI anchor. Therefore, a brand’s direct investment in an AI anchor not only proves most beneficial for all supply chain participants but also ensures the stability of the live streaming model.

**Observation 4.** *In scenarios with KOL Involvement,*

*(1)Increasing the KOL’s share of live streaming time enhances the likelihood of achieving a triple-win outcome, whereas increasing the regular anchor’s live streaming time may reduce this possibility.*

*(2) Introducing an AI anchor effectively enhances the stability of the live streaming model.*

Moreover, upon comparing Fig 5a and 5b, we observe that in scenarios involving KOL participation, increasing the KOL’s share of live streaming time enhances the probability of achieving a triple-win scenario among supply chain members. This enhancement is attributed to the KOL’s amplified role in decision-making and their typically superior ability to attract consumers and boost sales, which in turn improves the collective profitability of the supply chain. Conversely, in cases where KOLs are involved, increasing the regular anchor’s live streaming time tends to reduce the likelihood of such a favorable outcome. This is because the regular anchor often finds it challenging to assume a pivotal role in the presence of a dominant KOL, and allocating more live streaming time to them can result in a dilution of resources and a decrease in overall marketing effectiveness. Furthermore, while the introduction of an AI anchor might affect the interests of the KOL, it could also promote stability in the live streaming model. AI anchors contribute consistent performance, which helps mitigate operational uncertainties. When the brand opts to deploy an AI anchor, the KOL generally adjusts to this decision. At this juncture, irrespective of who finances the AI anchor, the brand’s decision uniformly influences both the KOL and the regular anchor. Consequently, once the brand determines the investor for the AI anchor, other members of the supply chain typically conform to this arrangement, accepting the decision as part of the operational strategy.

## 6 Conclusions

### 6.1 Concluding remarks and managerial implications

This study examines the impact of AI anchors on live streaming e-commerce supply chains, employing game theory to analyze anchor influence, live streaming time allocation, anchor effort levels, AI anchor intelligence levels, and investment models. As the live streaming industry matures, sales on live streaming platforms have gained traction among platforms and brands. However, traditional live streaming models (e.g., involving regular anchors or KOLs) have exhibited limitations. With advancements in AI technology, AI anchors have emerged as an innovative strategy, introducing a new business model to live streaming. Against this backdrop, this study compares different AI anchor investment models with and without KOL participation. The main findings and managerial implications are summarized as follows:

Firstly, from the perspective of brand owners, the introduction of AI anchors benefits them regardless of whether KOLs are involved or whether the AI anchor is invested in by the brand owner or the live streaming platform. For instance, during the 2023 “Double 11” period, brands like Philips and L’Oréal employed AI anchors for promotion, achieving remarkable results. According to a Philips representative, after launching AI anchors, the daily viewer count in the live streaming room repeatedly hit new highs, reaching nearly 80,000 viewers at its peak. For human anchors, in scenarios without KOL involvement, regardless of the AI anchor’s investment source, a significant level of influence can motivate regular anchors to increase their effort levels. However, in scenarios with KOL involvement, the introduction of AI anchors may lead to a decrease in the effort levels of both regular anchors and KOLs. It is noteworthy that the presence of KOLs does not alter a core result: the addition of AI anchors can negatively affect the core live streaming forces within the ecosystem. Specifically, without KOLs, AI anchors from any source may harm the interests of regular anchors. For example, JD Cloud’s AI anchor “Yan Xi” has high efficiency and 24/7 live streaming capabilities, which can weaken the role and bargaining power of regular anchors, leading to attrition or reduced income. In scenarios with KOLs, AI anchors might harm KOL interests. Interestingly, in scenarios with KOLs, AI anchors can have a positive effect only if regular anchor influence is relatively low. We recommend that brand owners actively consider introducing AI anchors in scenarios without KOL involvement to boost regular anchor motivation and overall live streaming effectiveness. In scenarios with KOL involvement, if regular anchor influence is low, introducing AI anchors can serve as a supplementary strategy. Additionally, brand owners should monitor potential negative impacts on core live streaming forces after AI anchor introduction, and ensure ecosystem balance and stability through careful planning and management.

Secondly, regardless of KOL involvement or the AI anchor’s investor, higher AI anchor influence is more beneficial for brand owners. For example, TTOUCHME selected a top-ranked real anchor in terms of fan base and sales as a prototype for creating an AI anchor, meticulously recording real live streaming scenarios to replicate the top anchor’s promotional prowess, resulting in over a 100% increase in Gross Merchandise Volume (GMV) through the AI anchor’s impactful performance. However, in scenarios with KOL involvement, when KOL influence exceeds a certain threshold, brand owner profits decrease as KOL influence increases. Moreover, adjusting the proportion of live streaming time across different anchors can yield varied outcomes in different live streaming modes. For instance, TTOUCHME achieved an additional 60% GMV growth by flexibly adjusting the live streaming duration of AI and real anchors in joint sessions. From the perspective of brand owners’ willingness to use AI anchors, in scenarios without KOL involvement, increasing the proportion of regular anchors’ live streaming time may weaken the motivation to introduce AI anchors. In scenarios with KOL involvement, increasing the proportion of KOL live streaming time can effectively reduce the likelihood of using AI anchors. Conversely, if the regular anchors’ live streaming time is increased, brand owners might seek to introduce AI anchors to supplement regular anchors’ deficiencies. We recommend that brand owners focus on enhancing AI anchor influence to maximize their benefits when introducing AI anchors. In scenarios with KOL involvement, they should closely monitor changes in KOL influence to prevent exceeding thresholds that lead to profit declines. Additionally, brand owners should flexibly adjust the live streaming time proportion of each anchor based on the characteristics of the live streaming mode to optimize live streaming outcomes and enhance the overall benefits of supply chain members. Notably, the successful collaboration between virtual idol “Luo Tianyi” and renowned KOL Li Jiaqi in a Taobao live streaming room serves as a successful example, setting a record of 6.3 million in popularity.

Thirdly, regardless of KOL involvement, brand owners benefit more from introducing AI anchors they have invested in themselves. This is a key reason why JD Home Appliances and JD Supermarket adopt AI anchors developed by JD Cloud’s Yan Xi team, achieving perfect live streaming results. For human anchors, if brand owners decide to introduce AI anchors, in scenarios without KOL involvement, AI anchors invested in by brand owners are more beneficial to regular anchors compared to those invested in by live streaming platforms. In scenarios with KOL involvement, introducing AI anchors, regardless of the investor, harms human anchors (including regular anchors and KOLs), with the negative impact being the same for both. Additionally, the study finds that in scenarios without KOLs, brand owners investing in AI anchors can better stimulate regular anchors’ effort levels and result in higher product prices. In scenarios with KOLs, AI anchors invested in by brand owners or live streaming platforms have the same impact on other supply chain members. It is noteworthy that regardless of KOL involvement, AI anchors invested in by brand owners exhibit advantages in intelligence levels over those invested in by live streaming platforms. For example, Perfect Diary introduced the virtual anchor “Stella,” developed using AI technology from Damo Academy, in 2020. Stella’s facial expressions, movements, and live streaming content can adjust in real-time according to the live streaming situation, demonstrating the flexibility and adaptability of AI anchors. We recommend that brand owners prioritize self-investment when considering AI anchors to maximize their benefits. Simultaneously, they should closely monitor the potential impacts of AI anchors on human anchors, especially in scenarios with KOL involvement, and actively seek strategies to balance the interests of all parties.

Fourthly, if AI anchors are not introduced, in scenarios without KOLs, achieving a “win-win” for supply chain members is possible only when regular anchors have a high proportion of live streaming time and strong influence. In scenarios with KOLs, a “triple-win” can only be achieved if regular anchors have a low proportion of live streaming time and both they and KOLs have strong influence. If brand owners decide to use AI anchors, regardless of KOL involvement, AI anchors invested in by brand owners benefit supply chain members, facilitating either a “win-win” or “triple-win” outcome. Interestingly, when brand owners decide to introduce AI anchors, despite potentially harming other anchors’ interests, it can enhance the stability of the live streaming mode. From the perspective of supply chain members’ preferences, increasing the proportion of live streaming time of the main forces in the live streaming mode can enhance the probability of achieving mutual benefits. We recommend that brand owners optimize anchor configuration and live streaming time arrangements to maximize benefits when not introducing AI anchors. When deciding to introduce AI anchors, they should prioritize self-investment and closely monitor the impact on live streaming mode stability. Additionally, brand owners should recognize that introducing AI anchors can enhance the stability of the live streaming mode and consider increasing the live streaming time proportion of major forces to boost the probability of mutual benefits.

### 6.2 Limitations and future research directions

The study has several limitations and potential extensions. Firstly, this study doesn’t considers the potential challenges that brand owners may face when integrating AI technology. Future research could delve into the technical development difficulties, consumer acceptance barriers, and ethical considerations that brand owners might encounter when introducing AI hosts, using questionnaire surveys as a method. Secondly, this paper primarily focuses on the application of AI anchors in the direct sales model of brand owners, neglecting the widely prevalent resale model in live streaming sales. Given the prevalence of the resale model in the live streaming e-commerce sector, it is important to investigate the operational mechanisms, efficiency, and market feedback of AI anchors within the resale model. Lastly, this paper assumes that anchors generally adopt a uniform retail pricing strategy within the same live streaming model. However, in real-world scenarios, different anchors often employ differentiated pricing strategies to attract consumers. Therefore, future research could explore the price competition behavior among anchors within the same live streaming model.

## Supporting information

S1 Text Appendices A and B. (PDF)
