# Supplementary material for: Will the inclusion of AI anchors enhance the operational performance of live streaming e-commerce supply chains?
Source: PLoS One. 2025 Jun 17;20(6):e0321995. doi: 10.1371/journal.pone.0321995 (PMC12173241; doi:10.1371/journal.pone.0321995)
Supplement: S1 Text — (PDF) [file pone.0321995.s001.pdf]

PLOS ONE

RESEARCH ARTICLE

Will the inclusion of AI anchors enhance the operational performance of live streaming e-commerce supply chains?

Xinyan Yan[3], **Qihui Lu**[1,2,3,4], **Di Xiao**[1,2,3,4]*

1 Modern Business Research Center of Zhejiang Gongshang University, Hangzhou, China, **2** Inamori Business School, Zhejiang Gongshang University, Hangzhou, China, **3** School of Business Administration (MBA), Zhejiang Gongshang University, Hangzhou, China, **4** Enterprise Digital Intelligence and Business Analysis Research Center, Zhejiang Gongshang University, Hangzhou, China

* xiaodi@mail.zjgsu.edu.cn

Abstract

With the rapid growth of the live streaming e-commerce market, traditional live streaming models are encountering mounting challenges, whereas the advent of artificial intelligence (AI) technology has breathed new life into live streaming. This paper delves into the role of the AI anchor and model selection in scenarios both with and without the involvement of the Key Opinion Leader (KOL). Specifically, the AI anchor, whether invested in by the brand or the live streaming platform, is integrated into the regular anchor model (Model NN) and the hybrid model combining the KOL and the regular anchor (Model NK). A series of Stackelberg game models are formulated to compare the sales effort level, AI intelligence level, anchor influence, and the proportion of live streaming time on live streaming e-commerce. The findings suggest that the AI anchor can effectively substitute for the human anchor under certain conditions, particularly when the brand controls the AI intelligence level. To further explore the impact of the AI anchor on decision-making and profits among supply chain participants, the paper offers a thorough analysis of decisions and profits in scenarios both with and without KOL involvement. The results disclose that, under suitable conditions, the incorporation of the AI anchor can substantially enhance the operational efficiency of live streaming e-commerce supply chains, leading to increased profitability for the brand. Notably, the allocation of live streaming time between different anchors in hybrid models significantly influences the profitability of supply chain members. This discovery provides crucial insights for brands in devising live streaming strategies.

Check for updates

OPEN ACCESS

Citation: Yan X, Lu Q, Xiao D (2025) Will the inclusion of AI anchors enhance the operational performance of live streaming e-commerce supply chains?. PLoS One 20(6): e0321995. https://doi.org/10.1371/journal.pone.0321995

Editor: Sudarsan Jayasingh, Sri Sivasubramaniya Nadar College of Engineering, INDIA

Received: October 21, 2024

Accepted: March 14, 2025

Published: June 17, 2025

Copyright: © 2025 Yan et al. This is an open access article distributed under the terms of the Creative Commons Attribution License, which permits unrestricted use, distribution, and reproduction in any medium, provided the original author and source are credited.

Data availability statement: All relevant data are within the manuscript.

Funding: This work is supported by the National Social Science Fund of China (21BGL109). The funders had no role in study design, data collection and analysis, decision to publish, or preparation of the manuscript.

Competing interests: The authors have declared that no competing interests exist.

S1 Appendix A

Table 5. Equilibrium outcomes and profits without KOL involvement.

Model NN	
$e_r^{NN} = \frac{\alpha_r \lambda_r d_0}{2n_r - 2\alpha_r \lambda_r^2}$	$p^{NN} = \frac{d_0 n_r}{2n_r - 2\alpha_r \lambda_r^2}$
$\pi_r^{NN} = \frac{(2n_r - 3\alpha_r \lambda_r^2)\alpha_r d_0^2 n_r}{2(2n_r - 2\alpha_r \lambda_r^2)^2}$	$\pi_b^{NN} = \frac{(1 - \alpha_r - \alpha_p) d_0^2 n_r}{2(2n_r - 2\alpha_r \lambda_r^2)}$
Model BN	
$e_r^{BN} = \frac{\delta \alpha_r \lambda_r (A+B) d_0 m}{2Am(n_r - \alpha_r \delta \lambda_r^2) + Bn_r(2m - B\lambda_a^2)}$	$s^{BN} = \frac{B\lambda_a (A+B) d_0 n_r}{2Am(n_r - \alpha_r \delta \lambda_r^2) + Bn_r[2m - B\lambda_a^2]}$
$p^{BN} = \frac{(A+B) d_0 m n_r}{2Am(n_r - \alpha_r \delta \lambda_r^2) + Bn_r(2m - B\lambda_a^2)}$	$\pi_r^{BN} = \frac{Am(2n_r - 3\delta\alpha_r \lambda_r^2) + B(2mn_r + \delta\alpha_r \lambda_r^2 m - 2B\lambda_a^2 n_r)}{2[2Am(n_r - \alpha_r \delta \lambda_r^2) + Bn_r(2m - B\lambda_a^2)]^2}(A+B)\alpha_r \delta d_0^2 m n_r$
$\pi_b^{BN} = \frac{(A+B) d_0^2 m n_r}{2[2Am(n_r - \alpha_r \delta \lambda_r^2) + Bn_r(2m - B\lambda_a^2)]^2}$	
Model PN	
$e_r^{PN} = \frac{\delta \alpha_r \lambda_r (A+B) d_0 m}{2Am(n_r - \delta\alpha_r \lambda_r^2) + 2Bn_r[m - (1-\delta)\alpha_p \lambda_a^2]}$	$s^{PN} = \frac{(1-\delta)\alpha_p \lambda_a (A+B) d_0 n_r}{2Am(n_r - \delta\alpha_r \lambda_r^2) + 2Bn_r[m - (1-\delta)\alpha_p \lambda_a^2]}$
$p^{PN} = \frac{(A+B) d_0 m n_r}{2Am(n_r - \delta\alpha_r \lambda_r^2) + 2Bn_r[m - (1-\delta)\alpha_p \lambda_a^2]}$	$\pi_r^{PN} = \frac{Am(2n_r - 3\delta\alpha_r \lambda_r^2) + B[2mn_r + \alpha_r \delta \lambda_r^2 m - 4(1-\delta)\alpha_p \lambda_a^2 n_r]}{2\{2Am(n_r - \delta\alpha_r \lambda_r^2) + 2Bn_r[m - (1-\delta)\alpha_p \lambda_a^2]\}^2}(A+B)\alpha_r \delta d_0^2 m n_r$
$\pi_b^{PN} = \frac{(A+B)^2 d_0^2 m n_r}{2\{2Am(n_r - \delta\alpha_r \lambda_r^2) + 2Bn_r[m - (1-\delta)\alpha_p \lambda_a^2]\}}$	$\pi_p^{PN} = \frac{A[2mn_r - 2\delta(2-\delta)\alpha_r \lambda_r^2 m + \alpha_p (1-\delta)^2 \lambda_a^2 n_r] + B[2mn_r - (1-\delta)(3+\delta)\alpha_p \lambda_a^2 n_r + \alpha_p^2 \lambda_a^2 n_r + 2\alpha_r \delta^2 \lambda_r^2 m]}{2\{2Am(n_r - \delta\alpha_r \lambda_r^2) + 2Bn_r[m - (1-\delta)\alpha_p \lambda_a^2]\}^2}(A+B)\alpha_p d_0^2 m n_r$

$A = (1 - \alpha_r - \alpha_p)\delta,\ B = (1 - \alpha_p)(1 - \delta)$

Table 6. Equilibrium outcomes and profits with KOL involvement.

**Model NK**	
$e_r^{NK} = \frac{\delta\alpha_r\lambda_r d_0 n_k}{n_r(2n_k - B\alpha_k\lambda_k{}^2)}$	$e_k^{NK} = \frac{(1-\delta)\alpha_k\lambda_k d_0}{2n_k-(1-\delta)\alpha_k\lambda_k{}^2}$
$p^{NK} = \frac{d_0 n_k}{2n_k-(1-\delta)\alpha_k\lambda_k{}^2}$	$\pi_r^{NK} = \frac{2n_r n_k+\delta\alpha_r\lambda_r{}^2 n_k-2(1-\delta)\alpha_k\lambda_k{}^2 n_r}{2n_r\left[2n_k-(1-\delta)\alpha_k\lambda_k{}^2\right]^2}\delta\alpha_r d_0{}^2 n_k$
$\pi_k^{NK} = \frac{(1-\delta)\alpha_k d_0{}^2 n_k}{2\left[2n_k-(1-\delta)\alpha_k\lambda_k{}^2\right]} + F$	$\pi_b^{NK} = \frac{\left[n_r n_k+\delta\alpha_r\lambda_r{}^2 n_k-(1-\delta)\alpha_k\lambda_k{}^2 n_r\right]An_k+B(1-\delta)n_r n_k{}^2}{n_r\left[2n_k-(1-\delta)\alpha_k\lambda_k{}^2\right]^2} d_0{}^2 - F$
**Models BK and PK**	
$e_r^{BK} = e_r^{PK} = \frac{\delta\alpha_r\lambda_r d_0 n_k}{n_r(2n_k-\mu\alpha_k\lambda_k{}^2)}$	$e_k^{BK} = e_k^{PK} = \frac{\mu\alpha_k\lambda_k d_0}{2n_k-\mu\alpha_k\lambda_k{}^2}$
$p^{BK} = p^{PK} = \frac{d_0 n_k}{2n_k-\mu\alpha_k\lambda_k{}^2}$	$\pi_r^{BK} = \pi_r^{PK} = \frac{2n_r n_k+\delta\alpha_r\lambda_r{}^2 n_k-2\mu\alpha_k\lambda_k{}^2 n_r}{2n_r(2n_k-\mu\alpha_k\lambda_k{}^2)^2}\delta\alpha_r d_0{}^2 n_k$
$\pi_k^{BK} = \pi_k^{PK} = \frac{\mu\alpha_k d_0{}^2 n_k}{2(2n_k-\mu\alpha_k\lambda_k{}^2)} + F$	
**Model BK**	
$s^{BK} = \frac{C\lambda_a d_0 n_k}{m(2n_k-\mu\alpha_k\lambda_k{}^2)}$	$\pi_b^{BK} = \frac{(A+B\mu+C)2n_r n_k m+2A\delta\alpha_r\lambda_r{}^2 n_k m-2(A+C)\mu\alpha_k\lambda_k{}^2 n_r m+C^2\lambda_a{}^2 n_r n_k}{2n_r m(2n_k-\mu\alpha_k\lambda_k{}^2)^2} d_0{}^2 n_k - F$
**Model PK**	
$s^{PK} = \frac{\alpha_p(1-\delta-\mu)\lambda_a d_0 n_k}{m(2n_k-\mu\alpha_k\lambda_k{}^2)}$	$\pi_b^{PK} = \frac{(A+B\mu+C)n_r n_k m+A\delta\alpha_r\lambda_r{}^2 n_k m+C(1-\delta-\mu)\alpha_p\lambda_a{}^2 n_r n_k-(A+C)\mu\alpha_k\lambda_k{}^2 n_r m}{n_r m(2n_k-\mu\alpha_k\lambda_k{}^2)^2} d_0{}^2 n_k - F$
$\pi_p^{PK} = \frac{2mn_r n_k-2(1-\mu)\mu\alpha_k\lambda_k{}^2 mn_r+2\delta^2\alpha_r\lambda_r{}^2 d_0 mn_k+(1-\delta-\mu)^2\alpha_p\lambda_a{}^2 n_r n_k}{2mn_r(2n_k-\mu\alpha_k\lambda_k{}^2)^2}\alpha_p d_0{}^2 n_k$	
$C = (1-\alpha_p)(1-\delta-\mu)$	

Table 7. Summary of thresholds used.

Thresholds	Description
λ_{r1}	$\sqrt{\frac{2[H\delta+(1-\alpha_p)(1-\delta)]+H(1-\alpha_p)(1-\delta)\lambda_a^2}{2\alpha_r[2H\delta+(1-\alpha_p)(1-\delta)]}}$
λ_{r2}	$\sqrt{\frac{[H\delta+(1-\alpha_p)(1-\delta)]\alpha_r+H(1-\alpha_p)(1-\delta)\alpha_p\lambda_a^2}{[2H\delta+(1-\alpha_p)(1-\delta)]\alpha_r(1-\alpha_p)}}$
λ_{r3}	$\sqrt{\frac{(1-\alpha_p)^2(1-\delta)\lambda_a^2[H(1+\delta)+3(1-\alpha_p)(1-\delta)]}{2H\delta\alpha_r\lambda_r^2[H\delta+(1-\alpha_p)(2-\delta)]}}$
λ_{r4}	$\sqrt{\frac{(1-\alpha_p)(1-\delta)\alpha_p\lambda_a^2[H(1+\delta)+(1-\alpha_p)(1-\delta)]}{H\delta\alpha_r[H\delta+(1-\alpha_p)(2-\delta)]}}$
λ_{r7}	$\sqrt{\frac{[H\delta+(1-\alpha_p)(1-\delta)]\alpha_r+(1-\alpha_p)^2H(1-\delta)\lambda_a^2}{2H(1-\alpha_p)\delta\alpha_r}}$
λ_{r8}	$\sqrt{\frac{\alpha_r+(1-\alpha_p)^2(1-\delta-\mu)\lambda_a^2-\alpha_r\mu\alpha_k\lambda_k^2}{2H\delta\alpha_r}}$
λ_{r9}	$\sqrt{\frac{\alpha_r+2(1-\alpha_p)(1-\delta-\mu)\alpha_p\lambda_a^2-\alpha_r\mu\alpha_k\lambda_k^2}{2H\delta\alpha_r}}$
λ_{r10}	$\sqrt{\frac{2[(1-\delta+\mu)-(1-\delta)\mu\alpha_k\lambda_k^2]\alpha_k\lambda_k^2}{[4-(1-\delta+\mu)\alpha_k\lambda_k^2]\delta\alpha_r}}$
λ_{k1}	$\sqrt{\frac{2(1-\alpha_k-\alpha_p)\mu+2H\delta^2\alpha_r\lambda_r^2+(1-\alpha_p)^2(1-\delta-\mu)^2\lambda_a^2}{[H\delta+(1-\alpha_p)(1-\delta-\mu)]\mu\alpha_k}}$
λ_{k2}	$\sqrt{\frac{2[(1-\alpha_k-\alpha_p)\mu+H\delta^2\alpha_r\lambda_r^2+(1-\alpha_p)(1-\delta-\mu)^2\alpha_p\lambda_a^2]}{[H\delta+(1-\alpha_p)(1-\delta-\mu)]\mu\alpha_k}}$
λ_{k3}	$\sqrt{\frac{\alpha_r+(1-\alpha_p)^2(1-\delta-\mu)\lambda_a^2}{\mu\alpha_r\alpha_k}}$
λ_{k4}	$\sqrt{\frac{\alpha_r+2(1-\alpha_p)(1-\delta-\mu)\alpha_p\lambda_a^2}{\mu\alpha_r\alpha_k}}$
λ_{a1}	$\sqrt{\frac{2[(1-\alpha_r-\alpha_p)\delta+(1-\alpha_p)(1-\delta)]+2(1-\alpha_p)\delta\alpha_r\lambda_r^2}{(1-\alpha_p)^2(1-\delta)}}$
λ_{a2}	$\sqrt{\frac{[(1-\alpha_r-\alpha_p)\delta+(1-\alpha_p)(1-\delta)]+(1-\alpha_p)\delta\alpha_r\lambda_r^2}{(1-\alpha_p)\alpha_p(1-\delta)}}$

$H = 1 - \alpha_r - \alpha_p$

https://doi.org/10.1371/journal.pone.0321995.t007

S2 Appendix B

B1 Derivation of the equilibrium outcomes in Model NN, Model BN and Model PN.

We use backward induction to solve the game , and obtain the equilibrium outcomes of Table 5. To ensure all the equilibrium outcomes are positive and rule out trivial results, we restrict parameters in the feasible parameter region $n_r - \alpha_r {\lambda_r}^2 > 0$, $m - (1-\delta)\alpha_p {\lambda_a}^2 > 0$.

B1.1 Model NN

In Model NN, the sequence of events is as follows: In Stage 1, the brand determines the retail price p^{NN}. In Stage 2, the regular anchor determines the effort level e_r^{NN}.

First, we obtain the optimal effort level from $\frac{\partial \pi_r^{NN}}{\partial e_r^{NN}} = 0$ as the following form:

$$e_r^{NN} = \frac{\alpha_r \lambda_r p^{NN}}{n_r} \tag{S1}$$

Then, substituting Eq S1 into π_b^{NN}, we can get the optimal retail price from $\frac{\partial \pi_b^{NN}}{\partial p^{NN}} = 0$ as the following form:

$$p^{NN} = \frac{d_0 n_r}{2n_r - 2\alpha_r {\lambda_r}^2} \tag{S2}$$

Finally, substituting Eqs S1 and S2 into Eqs 1 and 2 yields π_r^{NN} and π_b^{NN}.

B1.2 Model BN

In Model BN, the sequence of events is as follows: In Stage 1, the brand determines the retail price p^{BN} and the intelligence level s^{BN}. In Stage 2, the regular anchor determines the effort level e_r^{BN}.

First, we obtain the optimal effort level from $\frac{\partial \pi_r^{BN}}{\partial e_r^{BN}} = 0$ as the following form:

$$e_r^{BN} = \frac{\alpha_r \delta \lambda_r p^{BN}}{n_r} \tag{S3}$$

Then, substituting Eq S3 into π_b^{BN}, we can get the optimal intelligence level and retail price from $\frac{\partial \pi_b^{BN}}{\partial s^{BN}} = 0$and $\frac{\partial \pi_b^{NN}}{\partial p^{NN}} = 0$ as the following form:

$$s^{BN} = \frac{(1-\alpha_p)(1-\delta)\lambda_a\left[(1-\alpha_r-\alpha_p)\delta + (1-\alpha_p)(1-\delta)\right] d_0 n_r}{2(1-\alpha_r-\alpha_p)\delta m(n_r - \alpha_r \delta {\lambda_r}^2) + (1-\alpha_p)(1-\delta)n_r\left[2m - (1-\alpha_p)(1-\delta){\lambda_a}^2\right]} \tag{S4}$$

$$p^{BN} = \frac{\left[(1-\alpha_r-\alpha_p)\delta + (1-\alpha_p)(1-\delta)\right] d_0 m n_r}{2(1-\alpha_r-\alpha_p)\delta m(n_r - \alpha_r \delta {\lambda_r}^2) + (1-\alpha_p)(1-\delta)n_r\left[2m - (1-\alpha_p)(1-\delta){\lambda_a}^2\right]} \tag{S5}$$

Finally, substituting Eqs S3, S4 and S5 into Eqs 3 and 4 yields π_r^{BN} and π_b^{BN}.

B1.3 Model PN

In Model PN, the sequence of events is as follows: In Stage 1, the brand determines the retail price p^{PN}. In Stage 2, the live streaming platform determines the intelligence level s^{PN}. In Stage 3, the regular anchor determines the effort level e_r^{PN}.

First, we obtain the optimal effort level from $\frac{\partial \pi_r^{PN}}{\partial e_r^{PN}} = 0$ as the following form:

$$e_r^{PN} = \frac{\alpha_r \delta \lambda_r p^{PN}}{n_r} \tag{S6}$$

After, substituting Eq S6 into π_p^{PN}, we can get the optimal intelligence level from $\frac{\partial \pi_p^{PN}}{\partial s^{PN}} = 0$as the following form:

$$s^{PN} = \frac{\alpha_p (1-\delta) \lambda_a p^{PN}}{m} \tag{S7}$$

Then, substituting Eqs S6 and S7 into π_b^{PN}, we can get the optimal retail price from $\frac{\partial \pi_b^{PN}}{\partial p^{PN}} =$ 0as the following form:

$$p^{PN} = \frac{\left[(1-\alpha_r-\alpha_p)\delta + (1-\alpha_p)(1-\delta)\right] d_0 m n_r}{2(1-\alpha_r-\alpha_p)\delta m(n_r - \delta\alpha_r\lambda_r{}^2) + 2(1-\alpha_p)(1-\delta)n_r\left[m - (1-\delta)\alpha_p\lambda_a{}^2\right]} \tag{S8}$$

Finally, substituting Eqs S6, S7 and S8 into Eqs 5, 6 and 7 yields π_r^{PN}, π_p^{PN} and π_b^{PN}.

B2 To better focus on the research of this paper, we assume that $m = n_r = 1$.

Proof to Proposition 1: By compare π_b^{NN}, π_b^{BN} and π_b^{PN}, we have the following inequalities, which prove this proposition:

$\pi_b^{BN} - \pi_b^{NN} = \frac{(1-\alpha_r-\alpha_p)\{2(1-\alpha_r-\alpha_p)\delta(1-\alpha_r\delta\lambda_r{}^2)+(1-\alpha_p)(1-\delta)[2-(1-\alpha_p)(1-\delta)\lambda_a{}^2]\}d_0{}^2}{2(2-2\alpha_r\lambda_r{}^2)\{2(1-\alpha_r-\alpha_p)\delta(1-\alpha_r\delta\lambda_r{}^2)+(1-\alpha_p)(1-\delta)[2-(1-\alpha_p)(1-\delta)\lambda_a{}^2]\}} - \frac{[(1-\alpha_r-\alpha_p)\delta+(1-\alpha_p)(1-\delta)]^2(2-2\alpha_r\lambda_r{}^2)d_0{}^2}{2(2-2\alpha_r\lambda_r{}^2)\{2(1-\alpha_r-\alpha_p)\delta(1-\alpha_r\delta\lambda_r{}^2)+(1-\alpha_p)(1-\delta)[2-(1-\alpha_p)(1-\delta)\lambda_a{}^2]\}}$,

$\pi_b^{NN} - \pi_b^{PN} = \frac{(1-\alpha_r-\alpha_p)\{2(1-\alpha_r-\alpha_p)\delta(1-\delta\alpha_r\lambda_r{}^2)+2(1-\alpha_p)(1-\delta)[1-(1-\delta)\alpha_p\lambda_a{}^2]\}d_0{}^2}{2(2-2\alpha_r\lambda_r{}^2)\{2(1-\alpha_r-\alpha_p)\delta(1-\delta\alpha_r\lambda_r{}^2)+2(1-\alpha_p)(1-\delta)[1-(1-\delta)\alpha_p\lambda_a{}^2]\}} - \frac{[(1-\alpha_r-\alpha_p)\delta+(1-\alpha_p)(1-\delta)]^2(2-2\alpha_r\lambda_r{}^2)d_0{}^2}{2(2-2\alpha_r\lambda_r{}^2)\{2(1-\alpha_r-\alpha_p)\delta(1-\delta\alpha_r\lambda_r{}^2)+2(1-\alpha_p)(1-\delta)[1-(1-\delta)\alpha_p\lambda_a{}^2]\}}$. The signs of $\pi_b^{NN} - \pi_b^{BN}$ and $\pi_b^{NN} - \pi_b^{PN}$ depend on the numerator which increases in λ_r. It is easy to show that $\pi_b^{NN} - \pi_b^{BN} < 0$ for any $0 < \lambda_r < \sqrt{\frac{2[(1-\alpha_r-\alpha_p)\delta+(1-\alpha_p)(1-\delta)]+(1-\alpha_r-\alpha_p)(1-\alpha_p)(1-\delta)\lambda_a{}^2}{2\alpha_r[2(1-\alpha_r-\alpha_p)\delta+(1-\alpha_p)(1-\delta)]}}$ and $\pi_b^{NN} - \pi_b^{PN} < 0$ for any $0 < \lambda_r < \sqrt{\frac{[(1-\alpha_r-\alpha_p)\delta+(1-\alpha_p)(1-\delta)]\alpha_r+(1-\alpha_r-\alpha_p)(1-\alpha_p)(1-\delta)\alpha_p\lambda_a{}^2}{[2(1-\alpha_r-\alpha_p)\delta+(1-\alpha_p)(1-\delta)]\alpha_r(1-\alpha_p)}}$. □

Proof to Proposition 2: Using Eqs S2, S5 and S8, we have the following inequalities, which prove this lemma: $p^{NN} - p^{BN} = \frac{2(1-\alpha_r-\alpha_p)\delta(1-\delta\alpha_r\lambda_r{}^2)d_0+(1-\alpha_p)(1-\delta)[2-(1-\alpha_p)(1-\delta)\lambda_a{}^2]d_0}{2(1-\alpha_r\lambda_r{}^2)\{2(1-\alpha_r-\alpha_p)\delta(1-\delta\alpha_r\lambda_r{}^2)+(1-\alpha_p)(1-\delta)[2-(1-\alpha_p)(1-\delta)\lambda_a{}^2]\}} - \frac{2(1-\alpha_r\lambda_r{}^2)[(1-\alpha_r-\alpha_p)\delta+(1-\alpha_p)(1-\delta)]d_0}{2(1-\alpha_r\lambda_r{}^2)\{2(1-\alpha_r-\alpha_p)\delta(1-\delta\alpha_r\lambda_r{}^2)+(1-\alpha_p)(1-\delta)[2-(1-\alpha_p)(1-\delta)\lambda_a{}^2]\}}$,

$p^{NN} - p^{PN} = \frac{2(1-\alpha_r-\alpha_p)\delta(1-\delta\alpha_r\lambda_r{}^2)d_0+2(1-\alpha_p)(1-\delta)[1-\alpha_p(1-\delta)\lambda_a{}^2]d_0}{2(1-\alpha_r\lambda_r{}^2)\{2(1-\alpha_r-\alpha_p)\delta(1-\delta\alpha_r\lambda_r{}^2)+2(1-\alpha_p)(1-\delta)[1-(1-\alpha_p)(1-\delta)\lambda_a{}^2]\}} - \frac{2(1-\alpha_r\lambda_r{}^2)[(1-\alpha_r-\alpha_p)\delta+(1-\alpha_p)(1-\delta)]d_0}{2(1-\alpha_r\lambda_r{}^2)\{2(1-\alpha_r-\alpha_p)\delta(1-\delta\alpha_r\lambda_r{}^2)+2(1-\alpha_p)(1-\delta)[1-(1-\alpha_p)(1-\delta)\lambda_a{}^2]\}}$. The signs of $p^{NN}-p^{BN}$ and $p^{NN}-p^{PN}$ depend on the numerator which increases in λ_a. It is easy to show that $p^{NN}-p^{BN}<0$ for any $\sqrt{\frac{2[(1-\alpha_r-\alpha_p)\delta+(1-\alpha_p)]\alpha_r\lambda_r{}^2}{(1-\alpha_p)^2(1-\delta)}} < \lambda_a$ and $p^{NN}-p^{PN}<0$ for any $\sqrt{\frac{[(1-\alpha_r-\alpha_p)\delta+(1-\alpha_p)]\alpha_r\lambda_r{}^2}{(1-\alpha_p)(1-\delta)\alpha_p}} < \lambda_a$. Using Eqs S5 and S8, we can get $p^{BN} - p^{PN} = \frac{1}{2(1-\alpha_r-\alpha_p)\delta(1-\delta\alpha_r\lambda_r{}^2)+(1-\alpha_p)(1-\delta)[2-(1-\alpha_p)(1-\delta)\lambda_a{}^2]} \times \frac{(1-\alpha_p)(1-\delta)^2\lambda_a{}^2(1-3\alpha_p)[(1-\alpha_r-\alpha_p)\delta+(1-\alpha_p)(1-\delta)]d_0}{2(1-\alpha_r-\alpha_p)\delta(1-\delta\alpha_r\lambda_r{}^2)+2(1-\alpha_p)(1-\delta)[1-\alpha_p(1-\delta)\lambda_a{}^2]}$. □

Proof to Proposition 3: Using Eqs S1, S3 and S6, we have the following inequalities, which prove this lemma: $e_r^{NN} - e_r^{BN} = \frac{2(1-\alpha_r-\alpha_p)\delta(1-\delta\alpha_r\lambda_r^2)\alpha_r\lambda_r d_0+(1-\alpha_p)(1-\delta)\left[2-(1-\alpha_p)(1-\delta)\lambda_a^2\right]\alpha_r\lambda_r d_0}{(2-2\alpha_r\lambda_r^2)\left\{2(1-\alpha_r-\alpha_p)\delta(1-\delta\alpha_r\lambda_r^2)+(1-\alpha_p)(1-\delta)\left[2-(1-\alpha_p)(1-\delta)\lambda_a^2\right]\right\}} - \frac{2(1-\alpha_r\lambda_r^2)\left[(1-\alpha_r-\alpha_p)\delta+(1-\alpha_p)(1-\delta)\right]\delta\alpha_r\lambda_r d_0}{(2-2\alpha_r\lambda_r^2)\left\{2(1-\alpha_r-\alpha_p)\delta(1-\delta\alpha_r\lambda_r^2)+(1-\alpha_p)(1-\delta)\left[2-(1-\alpha_p)(1-\delta)\lambda_a^2\right]\right\}} > 0$,
$e_r^{NN} - e_r^{PN} = \frac{2(1-\alpha_r-\alpha_p)\delta(1-\delta\alpha_r\lambda_r^2)\alpha_r\lambda_r d_0+2(1-\alpha_p)(1-\delta)\left[1-\alpha_p(1-\delta)\lambda_a^2\right]\alpha_r\lambda_r d_0}{(2-2\alpha_r\lambda_r^2)\left\{2(1-\alpha_r-\alpha_p)\delta(1-\delta\alpha_r\lambda_r^2)+(1-\alpha_p)(1-\delta)\left[2-(1-\alpha_p)(1-\delta)\lambda_a^2\right]\right\}} - \frac{2(1-\alpha_r\lambda_r^2)\left[(1-\alpha_r-\alpha_p)\delta+(1-\alpha_p)(1-\delta)\right]\delta\alpha_r\lambda_r d_0}{(2-2\alpha_r\lambda_r^2)\left\{2(1-\alpha_r-\alpha_p)\delta(1-\delta\alpha_r\lambda_r^2)+(1-\alpha_p)(1-\delta)\left[2-(1-\alpha_p)(1-\delta)\lambda_a^2\right]\right\}} > 0$. □

Proof to Proposition 4: Using Eqs S4 and S7, we can get
$s^{BN} - s^{PN} = \frac{(1-\delta)\lambda_a\left[(1-\alpha_r-\alpha_p)\delta+(1-\alpha_p)(1-\delta)\right]d_0}{2(1-\alpha_r-\alpha_p)\delta(1-\delta\alpha_r\lambda_r^2)+(1-\alpha_p)(1-\delta)\left[2-(1-\alpha_p)(1-\delta)\lambda_a^2\right]} \times \frac{2(1-\alpha_r-\alpha_p)\delta(1-\delta\alpha_r\lambda_r^2)(1-\alpha_p)+2(1-\alpha_p)(1-\delta)\left[1-\alpha_p(1-\delta)\lambda_a^2\right](1-\alpha_p)}{2(1-\alpha_r-\alpha_p)\delta(1-\delta\alpha_r\lambda_r^2)+2(1-\alpha_p)(1-\delta)\left[1-\alpha_p(1-\delta)\lambda_a^2\right]} - \frac{(1-\delta)\lambda_a\left[(1-\alpha_r-\alpha_p)\delta+(1-\alpha_p)(1-\delta)\right]d_0}{2(1-\alpha_r-\alpha_p)\delta(1-\delta\alpha_r\lambda_r^2)+(1-\alpha_p)(1-\delta)\left[2-(1-\alpha_p)(1-\delta)\lambda_a^2\right]} \times \frac{2(1-\alpha_r-\alpha_p)\delta(1-\delta\alpha_r\lambda_r^2)\alpha_p+(1-\alpha_p)(1-\delta)\left[2-(1-\alpha_p)(1-\delta)\lambda_a^2\right]\alpha_p}{2(1-\alpha_r-\alpha_p)\delta(1-\delta\alpha_r\lambda_r^2)+2(1-\alpha_p)(1-\delta)\left[1-\alpha_p(1-\delta)\lambda_a^2\right]} > 0$. □

Proof to Proposition 5: By compare π_b^{BN} and π_b^{PN}, we have the following inequalities, which prove this proposition:
$\pi_b^{BN} - \pi_b^{PN} = \frac{1}{2\left\{2(1-\alpha_r-\alpha_p)\delta(1-\alpha_r\delta\lambda_r^2)+(1-\alpha_p)(1-\delta)\left[2-(1-\alpha_p)(1-\delta)\lambda_a^2\right]\right\}} \times \frac{\left[(1-\alpha_r-\alpha_p)\delta+(1-\alpha_p)(1-\delta)\right]^2 d_0^2(1-\alpha_p)(1-\delta)^2\lambda_a^2(1-3\alpha_p)}{2(1-\alpha_r-\alpha_p)\delta(1-\delta\alpha_r\lambda_r^2)+2(1-\alpha_p)(1-\delta)\left[1-(1-\delta)\alpha_p\lambda_a^2\right]} > 0$. □

Proof to Corollary 1: Taking the first-order condition of e_r^{BN}, s^{BN}, p^{BN} and π_b^{BN} with respect to λ_r, we can get
$\frac{\partial e_r^{BN}}{\partial \lambda_r} = \frac{\alpha_r\delta\left[(1-\alpha_r-\alpha_p)\delta+(1-\alpha_p)(1-\delta)\right]d_0\left\{2(1-\alpha_r-\alpha_p)\delta(1+\alpha_r\delta\lambda_r^2)+(1-\alpha_p)(1-\delta)\left[2-(1-\alpha_p)(1-\delta)\lambda_a^2\right]\right\}}{\left\{2(1-\alpha_r-\alpha_p)\delta(-\alpha_r\delta\lambda_r^2)+(1-\alpha_p)(1-\delta)\left[2-(1-\alpha_p)(1-\delta)\lambda_a^2\right]\right\}^2} > 0$,
$\frac{\partial s^{BN}}{\partial \lambda_r} = \frac{-(1-\alpha_p)(1-\delta)\lambda_a\left[(1-\alpha_r-\alpha_p)\delta+(1-\alpha_p)(1-\delta)\right]d_0\left\{2(1-\alpha_r-\alpha_p)\delta(-2\alpha_r\delta\lambda_r)\right\}}{\left\{2(1-\alpha_r-\alpha_p)\delta(1-\alpha_r\delta\lambda_r^2)+(1-\alpha_p)(1-\delta)\left[2-(1-\alpha_p)(1-\delta)\lambda_a^2\right]\right\}^2} > 0$,
$\frac{\partial p^{BN}}{\partial \lambda_r} = \frac{-\left[(1-\alpha_r-\alpha_p)\delta+(1-\alpha_p)(1-\delta)\right]d_0\left\{2(1-\alpha_r-\alpha_p)\delta(-2\alpha_r\delta\lambda_r)\right\}}{\left\{2(1-\alpha_r-\alpha_p)\delta(-\alpha_r\delta\lambda_r^2)+(1-\alpha_p)(1-\delta)\left[2-(1-\alpha_p)(1-\delta)\lambda_a^2\right]\right\}^2} > 0$,
$\frac{\partial \pi_b^{BN}}{\partial \lambda_r} = \frac{-\left[(1-\alpha_r-\alpha_p)\delta+(1-\alpha_p)(1-\delta)\right]^2 d_0^2 2\left\{2(1-\alpha_r-\alpha_p)\delta(-2\alpha_r\delta\lambda_r)\right\}}{4\left\{2(1-\alpha_r-\alpha_p)\delta(1-\alpha_r\delta\lambda_r^2)+(1-\alpha_p)(1-\delta)\left[2-(1-\alpha_p)(1-\delta)\lambda_a^2\right]\right\}^2} > 0$. Taking the first-order condition of e_r^{BN}, s^{BN}, p^{BN} and π_b^{BN} with respect to λ_a, we can get
$\frac{\partial e_r^{BN}}{\partial \lambda_a} = \frac{-\alpha_r\delta\lambda_r\left[(1-\alpha_r-\alpha_p)\delta+(1-\alpha_p)(1-\delta)\right]d_0\left\{(1-\alpha_p)(1-\delta)\left[-2(1-\alpha_p)(1-\delta)\lambda_a\right]\right\}}{\left\{2(1-\alpha_r-\alpha_p)\delta(1-\alpha_r\delta\lambda_r^2)+(1-\alpha_p)(1-\delta)n_r\left[2-(1-\alpha_p)(1-\delta)\lambda_a^2\right]\right\}^2} > 0$,
$\frac{\partial s^{BN}}{\partial \lambda_a} = \frac{(1-\alpha_p)(1-\delta)\left[(1-\alpha_r-\alpha_p)\delta+(1-\alpha_p)(1-\delta)\right]d_0\left\{2(1-\alpha_r-\alpha_p)\delta(1-\alpha_r\delta\lambda_r^2)+(1-\alpha_p)(1-\delta)\left[2+(1-\alpha_p)(1-\delta)\lambda_a^2\right]\right\}}{\left\{2(1-\alpha_r-\alpha_p)\delta(1-\alpha_r\delta\lambda_r^2)+(1-\alpha_p)(1-\delta)\left[2-(1-\alpha_p)(1-\delta)\lambda_a^2\right]\right\}^2} > 0$,
$\frac{\partial p^{BN}}{\partial \lambda_a} = \frac{-\left[(1-\alpha_r-\alpha_p)\delta+(1-\alpha_p)(1-\delta)\right]d_0(1-\alpha_p)(1-\delta)\left[-2(1-\alpha_p)(1-\delta)\lambda_a\right]}{\left\{2(1-\alpha_r-\alpha_p)\delta(1-\alpha_r\delta\lambda_r^2)+(1-\alpha_p)(1-\delta)\left[2-(1-\alpha_p)(1-\delta)\lambda_a^2\right]\right\}^2} > 0$,
$\frac{\partial \pi_b^{BN}}{\partial \lambda_a} = \frac{-\left[(1-\alpha_r-\alpha_p)\delta+(1-\alpha_p)(1-\delta)\right]^2 d_0^2 2(1-\alpha_p)(1-\delta)\left[-2(1-\alpha_p)(1-\delta)\lambda_a\right]}{4\left\{2(1-\alpha_r-\alpha_p)\delta(1-\alpha_r\delta\lambda_r^2)+(1-\alpha_p)(1-\delta)\left[2-(1-\alpha_p)(1-\delta)\lambda_a^2\right]\right\}^2} > 0$. Taking the first-order condition of e_r^{PN}, s^{PN}, p^{PN} and π_b^{PN} with respect to λ_r, we can get
$\frac{\partial e_r^{PN}}{\partial \lambda_r} = \frac{\alpha_r\delta\left[(1-\alpha_r-\alpha_p)\delta+(1-\alpha_p)(1-\delta)\right]d_0\left\{2(1-\alpha_r-\alpha_p)\delta(1+\delta\alpha_r\lambda_r^2)+2(1-\alpha_p)(1-\delta)\left[1-(1-\delta)\alpha_p\lambda_a^2\right]\right\}}{\left\{2(1-\alpha_r-\alpha_p)\delta(1-\delta\alpha_r\lambda_r^2)+2(1-\alpha_p)(1-\delta)\left[1-(1-\delta)\alpha_p\lambda_a^2\right]\right\}^2} > 0$,
$\frac{\partial s^{PN}}{\partial \lambda_r} = \frac{-\alpha_p(1-\delta)\lambda_a\left[(1-\alpha_r-\alpha_p)\delta+(1-\alpha_p)(1-\delta)\right]d_0 2(1-\alpha_r-\alpha_p)\delta(-2\delta\alpha_r\lambda_r)}{\left\{2(1-\alpha_r-\alpha_p)\delta(1-\delta\alpha_r\lambda_r^2)+2(1-\alpha_p)(1-\delta)\left[1-(1-\delta)\alpha_p\lambda_a^2\right]\right\}^2} > 0$,
$\frac{\partial p^{PN}}{\partial \lambda_r} = \frac{-\left[(1-\alpha_r-\alpha_p)\delta+(1-\alpha_p)(1-\delta)\right]d_0 2(1-\alpha_r-\alpha_p)\delta(-\delta\alpha_r\lambda_r^2)}{\left\{2(1-\alpha_r-\alpha_p)\delta(1-\delta\alpha_r\lambda_r^2)+2(1-\alpha_p)(1-\delta)\left[1-(1-\delta)\alpha_p\lambda_a^2\right]\right\}^2} > 0$,

$\frac{\partial \pi_b^{PN}}{\partial \lambda_r} = \frac{-[(1-\alpha_r-\alpha_p)\delta+(1-\alpha_p)(1-\delta)]^2 d_0{}^2 2\{2(1-\alpha_r-\alpha_p)\delta(-2\delta\alpha_r\lambda_r)\}}{4\{2(1-\alpha_r-\alpha_p)\delta(1-\delta\alpha_r\lambda_r{}^2)+2(1-\alpha_p)(1-\delta)[1-(1-\delta)\alpha_p\lambda_a{}^2]\}^2} > 0$. Taking the first-order condition of e_r^{PN}, s^{PN}, p^{PN} and π_b^{PN} with respect to λ_a, we can get

$\frac{\partial e_r^{PN}}{\partial \lambda_a} = \frac{-\alpha_r\delta\lambda_r[(1-\alpha_r-\alpha_p)\delta+(1-\alpha_p)(1-\delta)]d_0 2(1-\alpha_p)(1-\delta)[-(1-\delta)\alpha_p\lambda_a{}^2]}{\{2(1-\alpha_r-\alpha_p)\delta(1-\delta\alpha_r\lambda_r{}^2)+2(1-\alpha_p)(1-\delta)[1-(1-\delta)\alpha_p\lambda_a{}^2]\}^2} > 0$,

$\frac{\partial s^{PN}}{\partial \lambda_a} = \frac{\alpha_p(1-\delta)[(1-\alpha_r-\alpha_p)\delta+(1-\alpha_p)(1-\delta)]d_0\{2(1-\alpha_r-\alpha_p)\delta(1-\delta\alpha_r\lambda_r{}^2)+2(1-\alpha_p)(1-\delta)[1+(1-\delta)\alpha_p\lambda_a{}^2]\}}{\{2(1-\alpha_r-\alpha_p)\delta(1-\delta\alpha_r\lambda_r{}^2)+2(1-\alpha_p)(1-\delta)[1-(1-\delta)\alpha_p\lambda_a{}^2]\}^2} > 0$,

$\frac{\partial p^{PN}}{\partial \lambda_a} = \frac{-[(1-\alpha_r-\alpha_p)\delta+(1-\alpha_p)(1-\delta)]d_0\{2(1-\alpha_p)(1-\delta)[-(1-\delta)\alpha_p\lambda_a{}^2]\}}{\{2(1-\alpha_r-\alpha_p)\delta(1-\delta\alpha_r\lambda_r{}^2)+2(1-\alpha_p)(1-\delta)[1-(1-\delta)\alpha_p\lambda_a{}^2]\}^2} > 0$,

$\frac{\partial \pi_b^{PN}}{\partial \lambda_a} = \frac{-[(1-\alpha_r-\alpha_p)\delta+(1-\alpha_p)(1-\delta)]^2 d_0{}^2 2\{2(1-\alpha_p)(1-\delta)[-2(1-\delta)\alpha_p\lambda_a]\}}{4\{2(1-\alpha_r-\alpha_p)\delta(1-\delta\alpha_r\lambda_r{}^2)+2(1-\alpha_p)(1-\delta)[1-(1-\delta)\alpha_p\lambda_a{}^2]\}^2} > 0$. □

Proof to Corollary 2: Taking the first-order condition of e_r^{BN}, e_r^{PN}, s^{BN}, s^{PN}, p^{BN}, p^{PN} and π_b^{BN} with respect to δ, we can get $\frac{\partial e_r^{BN}}{\partial \delta} = [(1-\alpha_r-\alpha_p)2\delta + (1-\alpha_p)(1-2\delta)]\alpha_r\lambda_r d_0 \times \frac{2(1-\alpha_r-\alpha_p)\delta(1-\alpha_r\delta\lambda_r{}^2)+(1-\alpha_p)(1-\delta)[2-(1-\alpha_p)(1-\delta)\lambda_a{}^2]}{\{2(1-\alpha_r-\alpha_p)\delta(1-\alpha_r\delta\lambda_r{}^2)+(1-\alpha_p)(1-\delta)[2-(1-\alpha_p)(1-\delta)\lambda_a{}^2]\}^2} - \delta[(1-\alpha_r-\alpha_p)\delta + (1-\alpha_p)(1-\delta)]\alpha_r\lambda_r d_0 \times \frac{2(1-\alpha_r-\alpha_p)(1-2\alpha_r\delta\lambda_r{}^2)+(1-\alpha_p)[-2+(1-\alpha_p)2(1-\delta)\lambda_a{}^2]}{\{2(1-\alpha_r-\alpha_p)\delta(1-\alpha_r\delta\lambda_r{}^2)+(1-\alpha_p)(1-\delta)[2-(1-\alpha_p)(1-\delta)\lambda_a{}^2]\}^2} > 0$,

$\frac{\partial e_r^{PN}}{\partial \delta} = [(1-\alpha_r-\alpha_p)2\delta + (1-\alpha_p)(1-2\delta)]\alpha_r\lambda_r d_0 \times \frac{2(1-\alpha_r-\alpha_p)\delta(1-\delta\alpha_r\lambda_r{}^2)+2(1-\alpha_p)(1-\delta)[1-(1-\delta)\alpha_p\lambda_a{}^2]}{\{2(1-\alpha_r-\alpha_p)\delta(1-\delta\alpha_r\lambda_r{}^2)+2(1-\alpha_p)(1-\delta)[1_r-(1-\delta)\alpha_p\lambda_a{}^2]\}^2} - \delta[(1-\alpha_r-\alpha_p)\delta + (1-\alpha_p)(1-\delta)]\alpha_r\lambda_r d_0 \times \frac{2(1-\alpha_r-\alpha_p)(1-2\delta\alpha_r\lambda_r{}^2)+2(1-\alpha_p)[-1+2(1-\delta)\alpha_p\lambda_a{}^2]}{\{2(1-\alpha_r-\alpha_p)\delta(1-\delta\alpha_r\lambda_r{}^2)+2(1-\alpha_p)(1-\delta)[1_r-(1-\delta)\alpha_p\lambda_a{}^2]\}^2} > 0$,

$\frac{\partial s^{BN}}{\partial \delta} = [(1-\alpha_r-\alpha_p)(1-2\delta) - (1-\alpha_p)2(1-\delta)](1-\alpha_p)\lambda_a d_0 \times \frac{2(1-\alpha_r-\alpha_p)\delta(1-\delta\alpha_r\lambda_r{}^2)+(1-\alpha_p)(1-\delta)[2-(1-\alpha_p)(1-\delta)\lambda_a{}^2]}{\{2(1-\alpha_r-\alpha_p)\delta(1-\alpha_r\delta\lambda_r{}^2)+(1-\alpha_p)(1-\delta)[2-(1-\alpha_p)(1-\delta)\lambda_a{}^2]\}^2} - (1-\delta)[(1-\alpha_r-\alpha_p)\delta + (1-\alpha_p)(1-\delta)](1-\alpha_p)\lambda_a d_0 \times \frac{2(1-\alpha_r-\alpha_p)(1-2\delta\alpha_r\lambda_r{}^2)+(1-\alpha_p)[-2+(1-\alpha_p)2(1-\delta)\lambda_a{}^2]}{\{2(1-\alpha_r-\alpha_p)\delta(1-\alpha_r\delta\lambda_r{}^2)+(1-\alpha_p)(1-\delta)[2-(1-\alpha_p)(1-\delta)\lambda_a{}^2]\}^2} < 0$,

$\frac{\partial s^{PN}}{\partial \delta} = 2[(1-\alpha_r-\alpha_p)(1-2\delta) - (1-\alpha_p)2(1-\delta)]\alpha_p\lambda_a d_0 \times \frac{(1-\alpha_r-\alpha_p)\delta(1-\delta\alpha_r\lambda_r{}^2)+(1-\alpha_p)(1-\delta)[1-(1-\delta)\alpha_p\lambda_a{}^2]}{\{2(1-\alpha_r-\alpha_p)\delta(1-\delta\alpha_r\lambda_r{}^2)+2(1-\alpha_p)(1-\delta)[1-(1-\delta)\alpha_p\lambda_a{}^2]\}^2} - 2(1-\delta)[(1-\alpha_r-\alpha_p)\delta + (1-\alpha_p)(1-\delta)]\alpha_p\lambda_a d_0 \times \frac{(1-\alpha_r-\alpha_p)(1-2\delta\alpha_r\lambda_r{}^2)+(1-\alpha_p)[-1+2(1-\delta)\alpha_p\lambda_a{}^2]}{\{2(1-\alpha_r-\alpha_p)\delta(1-\delta\alpha_r\lambda_r{}^2)+2(1-\alpha_p)(1-\delta)[1-(1-\delta)\alpha_p\lambda_a{}^2]\}^2} < 0$,

$\frac{\partial p^{BN}}{\partial \delta} = [(1-\alpha_r-\alpha_p) - (1-\alpha_p)]d_0 \times \frac{2(1-\alpha_r-\alpha_p)\delta(1-\alpha_r\delta\lambda_r{}^2)+(1-\alpha_p)(1-\delta)[2-(1-\alpha_p)(1-\delta)\lambda_a{}^2]}{\{2(1-\alpha_r-\alpha_p)\delta(1-\alpha_r\delta\lambda_r{}^2)+(1-\alpha_p)(1-\delta)[2-(1-\alpha_p)(1-\delta)\lambda_a{}^2]\}^2} - [(1-\alpha_r-\alpha_p)\delta + (1-\alpha_p)(1-\delta)]d_0 \times \frac{2(1-\alpha_r-\alpha_p)(1-2\delta\alpha_r\lambda_r{}^2)+(1-\alpha_p)[-2+(1-\alpha_p)2(1-\delta)\lambda_a{}^2]}{\{2(1-\alpha_r-\alpha_p)\delta(1-\alpha_r\delta\lambda_r{}^2)+(1-\alpha_p)(1-\delta)[2-(1-\alpha_p)(1-\delta)\lambda_a{}^2]\}^2}$,

$\frac{\partial p^{PN}}{\partial \delta} = 2[(1-\alpha_r-\alpha_p) - (1-\alpha_p)]d_0 \times \frac{(1-\alpha_r-\alpha_p)\delta(1-\delta\alpha_r\lambda_r{}^2)+(1-\alpha_p)(1-\delta)[1-(1-\delta)\alpha_p\lambda_a{}^2]}{\{2(1-\alpha_r-\alpha_p)\delta(1-\delta\alpha_r\lambda_r{}^2)+2(1-\alpha_p)(1-\delta)[1-(1-\delta)\alpha_p\lambda_a{}^2]\}^2} - 2[(1-\alpha_r-\alpha_p)\delta + (1-\alpha_p)(1-\delta)]d_0 \times \frac{(1-\alpha_r-\alpha_p)(1-2\delta\alpha_r\lambda_r{}^2)+(1-\alpha_p)[-1+2(1-\delta)\alpha_p\lambda_a{}^2]}{\{2(1-\alpha_r-\alpha_p)\delta(1-\delta\alpha_r\lambda_r{}^2)+2(1-\alpha_p)(1-\delta)[1-(1-\delta)\alpha_p\lambda_a{}^2]\}^2}$,

$\frac{\partial \pi_b^{BN}}{\partial \delta} = \frac{[(1-\alpha_r-\alpha_p)\delta+(1-\alpha_p)(1-\delta)][(1-\alpha_r-\alpha_p)-(1-\alpha_p)]d_0{}^2}{2(1-\alpha_r-\alpha_p)\delta(1-\alpha_r\delta\lambda_r{}^2)+(1-\alpha_p)(1-\delta)[2-(1-\alpha_p)(1-\delta)\lambda_a{}^2]} - [(1-\alpha_r-\alpha_p)\delta + (1-\alpha_p)(1-\delta)]^2 d_0{}^2 \times \frac{(1-\alpha_r-\alpha_p)(1-2\alpha_r\delta\lambda_r{}^2)+(1-\alpha_p)[-1+(1-\alpha_p)(1-\delta)\lambda_a{}^2]}{\{2(1-\alpha_r-\alpha_p)\delta(1-\alpha_r\delta\lambda_r{}^2)+(1-\alpha_p)(1-\delta)[2-(1-\alpha_p)(1-\delta)\lambda_a{}^2]\}^2}$.

The signs of $\frac{\partial p^{BN}}{\partial \delta}$, $\frac{\partial p^{PN}}{\partial \delta}$ and $\frac{\partial \pi_b^{BN}}{\partial \delta}$ depend on the numerator which increases in λ_r. It is easy to show that $\frac{\partial p^{BN}}{\partial \delta} < 0$ for any $0 < \lambda_r < \sqrt{\frac{(1-\alpha_p)^2(1-\delta)\lambda_a{}^2[(1-\alpha_r-\alpha_p)(1+\delta)+3(1-\alpha_p)(1-\delta)]}{2(1-\alpha_r-\alpha_p)\delta\alpha_r\lambda_r{}^2[(1-\alpha_r-\alpha_p)\delta+(1-\alpha_p)(2-\delta)]}}$, $\frac{\partial p^{PN}}{\partial \delta} < 0$ for any $0 < \lambda_r < \sqrt{\frac{(1-\alpha_p)(1-\delta)\alpha_p\lambda_a{}^2[(1-\alpha_r-\alpha_p)(1+\delta)+(1-\alpha_p)(1-\delta)]}{(1-\alpha_r-\alpha_p)\delta\alpha_r[(1-\alpha_r-\alpha_p)\delta+(1-\alpha_p)(2-\delta)]}}$, $\frac{\partial \pi_b^{BN}}{\partial \delta} < 0$ for any

$0 < \lambda_r < \sqrt{\frac{[(1-\alpha_r-\alpha_p)\delta+(1-\alpha_p)(1-\delta)]\alpha_r+(1-\alpha_p)^2(1-\alpha_r-\alpha_p)(1-\delta)\lambda_a^2}{2(1-\alpha_r-\alpha_p)(1-\alpha_p)\delta\alpha_r}}$. Taking the first-order condition of π_b^{PN} with respect to δ, we can get
$\frac{\partial \pi_b^{PN}}{\partial \delta} = \frac{[(1-\alpha_r-\alpha_p)\delta+(1-\alpha_p)(1-\delta)][(1-\alpha_r-\alpha_p)-(1-\alpha_p)]d_0^2}{2(1-\alpha_r-\alpha_p)\delta(1-\delta\alpha_r\lambda_r^2)+2(1-\alpha_p)(1-\delta)[1-(1-\delta)\alpha_p\lambda_a^2]} - [(1-\alpha_r-\alpha_p)\delta+(1-\alpha_p)(1-\delta)]^2 d_0^2 \times \frac{(1-\alpha_r-\alpha_p)(1-2\delta\alpha_r\lambda_r^2)+(1-\alpha_p)[-1+2(1-\delta)\alpha_p\lambda_a^2]}{\{2(1-\alpha_r-\alpha_p)\delta(1-\delta\alpha_r\lambda_r^2)+2(1-\alpha_p)(1-\delta)[1-(1-\delta)\alpha_p\lambda_a^2]\}^2} < 0.$ □

B3 Derivation of the equilibrium outcomes in Model NK, Model BK and Model PK.

We use backward induction to solve the game , and obtain the equilibrium outcomes of Table 6. To ensure all the equilibrium outcomes are positive and rule out trivial results, we restrict parameters in the feasible parameter region $2n_k - (1-\delta)\alpha_k\lambda_k^2 > 0$, $2n_k - \mu\alpha_k\lambda_k^2 > 0$.

B3.1 Model NK

In Model NK, the sequence of events is as follows: In Stage 1, the KOL determines the retail price p^{NK} and the effort level e_k^{NK}. In Stage 2, the regular anchor determines the effort level e_r^{NN}.

First, we obtain the optimal effort level from $\frac{\partial \pi_r^{NK}}{\partial e_r^{NK}} = 0$ as the following form:

$$e_r^{NK} = \frac{\delta\alpha_r\lambda_r p^{NK}}{n_r} \tag{S9}$$

Then, substituting Eq S9 into π_k^{NK}, we can get the optimal effort level and retail price from $\frac{\partial \pi_k^{NK}}{\partial e_k^{NK}} = 0$ and $\frac{\partial \pi_k^{NK}}{\partial p^{NK}} = 0$ as the following form:

$$e_k^{NK} = \frac{(1-\delta)\alpha_k\lambda_k d_0}{2n_k - (1-\delta)\alpha_k\lambda_k^2} \tag{S10}$$

$$p^{NK} = \frac{d_0 n_k}{2n_k - (1-\delta)\alpha_k\lambda_k^2} \tag{S11}$$

Finally, substituting Eqs S9, S10 and S11 into Eqs 8, 9 and 10 yields π_r^{NK}, $\pi_k^{NK}$and π_b^{NK}.

B3.2 Model BK

In Model BK, the sequence of events is as follows: In Stage 1, the KOL determines the retail price p^{BK} and the effort level e_k^{BK}. In Stage 2, the brand determines the intelligence level s^{BK}. In Stage 3, the regular anchor determines the effort level e_r^{BK}.

First, we obtain the optimal effort level from $\frac{\partial \pi_r^{BK}}{\partial e_r^{BK}} = 0$ as the following form:

$$e_r^{BK} = \frac{\delta\alpha_r\lambda_r p^{BK}}{n_r} \tag{S12}$$

After, substituting Eq S12 into π_b^{BK}, we can get the optimal intelligence level from $\frac{\partial \pi_b^{BK}}{\partial s^{BK}} = 0$ as the following form:

$$e_r^{BK} = \frac{\delta\alpha_r\lambda_r p^{BK}}{n_r} \tag{S13}$$

Then, substituting Eq S12 and S13 into π_k^{BK}, we can get the optimal effort level and retail price from $\frac{\partial \pi_k^{BK}}{\partial e_k^{BK}} = 0$ and $\frac{\partial \pi_k^{BK}}{\partial p^{BK}} = 0$ as the following form:

$$e_k^{BK} = \frac{\mu \alpha_k \lambda_k d_0}{2n_k - \mu \alpha_k {\lambda_k}^2} \tag{S14}$$

$$p^{BK} = \frac{d_0 n_k}{2n_k - \mu \alpha_k {\lambda_k}^2} \tag{S15}$$

Finally, substituting Eqs 12, S13, S14 and S15 into Eqs 11, 12 and 13 yields π_r^{BK}, π_k^{BK} and π_b^{BK}.

B3.3 Model PK

In Model PK, the sequence of events is as follows: In Stage 1, the KOL determines the retail price p^{PK} and the effort level e_k^{PK}. In Stage 2, the live streaming platform determines the intelligence level s^{PK}. In Stage 3, the regular anchor determines the effort level e_r^{PK}.

First, we obtain the optimal effort level from $\frac{\partial \pi_r^{PK}}{\partial e_r^{PK}} = 0$ as the following form:

$$e_r^{PK} = \frac{\delta \alpha_r \lambda_r p^{PK}}{n_r} \tag{S16}$$

After, substituting Eq 16 into π_p^{PK}, we can get the optimal intelligence level from $\frac{\partial \pi_p^{PK}}{\partial s^{PK}} = 0$as the following form:

$$s^{PK} = \frac{(1 - \delta - \mu)\alpha_p \lambda_a p^{PK}}{m} \tag{S17}$$

Then, substituting Eq S16 and S17 into π_k^{PK}, we can get the optimal effort level and retail price from $\frac{\partial \pi_k^{PK}}{\partial e_k^{PK}} = 0$ and $\frac{\partial \pi_k^{PK}}{\partial p^{PK}} = 0$ as the following form:

$$e_k^{PK} = \frac{\mu \alpha_k \lambda_k d_0}{2n_k - \mu \alpha_k {\lambda_k}^2} \tag{S18}$$

$$p^{PK} = \frac{d_0 n_k}{2n_k - \mu \alpha_k {\lambda_k}^2} \tag{S19}$$

Finally, substituting Eqs S16, S17, S18 and S19 into Eqs14, 15, 16 and 17 yields π_r^{PK}, π_k^{PK}, π_p^{PK} and π_b^{PK}.

B4 To better focus on the research of this paper, we assume that $m = n_r = n_k = 1$.

Proof to Proposition 6: Using Eqs S11, S15 and S19, we can get $p^{NK} - p^{BK} = p^{NK} - p^{PK} = \frac{(1-\delta-\mu)\alpha_k {\lambda_k}^2 d_0}{\left[2-(1-\delta)\alpha_k {\lambda_k}^2\right](2-\mu\alpha_k\lambda_k^2)} > 0$. Using Eqs S9, S12 and S16, we can get $e_r^{NK} - e_r^{BK} = e_r^{NK} - e_r^{PK} = \delta\alpha_r\lambda_r(p^{NK} - p^{PK}) > 0$. Using Eqs S10, S14 and S18, we can get $e_k^{NK} - e_k^{BK} = e_k^{NK} - e_k^{PK} = \frac{2\alpha_k\lambda_k(1-\delta-\mu)d_0}{\left[2-(1-\delta)\alpha_k\lambda_k^2\right](2-\mu\alpha_k\lambda_k^2)} > 0$. Using Eqs S13 and S17, we can get $s^{BK} - s^{PK} = (1 - \delta - \mu)\lambda_a p^{BK}(1 - 2\alpha_p) > 0$. □

Proof to Proposition 7: By compare π_b^{BK} and π_b^{PK}, we have the following inequalities, which prove this proposition: $\pi_b^{BK} - \pi_b^{PK} = \frac{(1-\alpha_p)^2(1-\delta-\mu)^2\lambda_a{}^2(1-3\alpha_p)}{2(2-\mu\alpha_k\lambda_k{}^2)^2} > 0$. □

Proof to Proposition 8: By compare π_r^{NK}, π_r^{BK} and π_r^{PK}, we have the following inequalities, which prove this proposition: $\pi_r^{NK} - \pi_r^{BK} = \pi_r^{NK} - \pi_r^{PK} = \delta\alpha_r d_0{}^2 \times \frac{[2+\delta\alpha_r\lambda_r{}^2-2(1-\delta)\alpha_k\lambda_k{}^2](2-\mu\alpha_k\lambda_k{}^2)^2}{2[2-(1-\delta)\alpha_k\lambda_k{}^2]^2(2-\mu\alpha_k\lambda_k{}^2)^2} - \delta\alpha_r d_0{}^2 \times \frac{(2+\delta\alpha_r\lambda_r{}^2-2\mu\alpha_k\lambda_k{}^2)[2-(1-\delta)\alpha_k\lambda_k{}^2]^2}{2[2-(1-\delta)\alpha_k\lambda_k{}^2]^2(2-\mu\alpha_k\lambda_k{}^2)^2}$. The signs of $\pi_r^{NK} - \pi_r^{BK}$ or $\pi_r^{NK} - \pi_r^{PK}$ depend on the numerator which increases in λ_r. It is easy to show that $\pi_b^{NN} - \pi_b^{BN} > 0$ or $\pi_r^{NK} - \pi_r^{PK} > 0$ for any $\sqrt{\frac{2[(1-\delta+\mu)-(1-\delta)\mu\alpha_k\lambda_k{}^2]\alpha_k\lambda_k{}^2}{[4-(1-\delta+\mu)\alpha_k\lambda_k{}^2]\delta\alpha_r}} < \lambda_r$. By compare π_k^{NK}, π_k^{BK} and π_k^{PK}, we have the following inequalities, which prove this proposition: $\pi_k^{NK} - \pi_k^{BK} = \pi_k^{NK} - \pi_k^{PK} = \alpha_k d_0{}^2 \frac{(1-\delta)(2-\mu\alpha_k\lambda_k{}^2)-\mu[2-(1-\delta)\alpha_k\lambda_k{}^2]}{2[2-(1-\delta)\alpha_k\lambda_k{}^2](2-\mu\alpha_k\lambda_k{}^2)} > 0$. □

Proof to Proposition 9: By compare π_r^{BK} and π_r^{PK}, we have the following inequalities, which prove this proposition: $\pi_r^{BK} - \pi_r^{PK} = 0$. By compare π_k^{BK} and π_k^{PK}, we have the following inequalities, which prove this proposition: $\pi_k^{BK} - \pi_k^{PK} = 0$. □

Proof to Corollary 3: Taking the first-order condition of e_r^{BK} and π_b^{BK} with respect to λ_r, we can get $\frac{\partial e_r^{BK}}{\partial\lambda_r} = \frac{\delta\alpha_r d_0}{2-\mu\alpha_k\lambda_k{}^2} > 0$, $\frac{\partial\pi_b^{BK}}{\partial\lambda_r} = \frac{4(1-\alpha_r-\alpha_p)\delta^2\alpha_r\lambda_r}{2(2-\mu\alpha_k\lambda_k{}^2)^2} d_0{}^2 > 0$. Taking the first-order condition of s^{BK} and π_b^{BK} with respect to λ_a, we can get $\frac{\partial s^{BK}}{\partial\lambda_a} = \frac{(1-\alpha_p)(1-\delta-\mu)d_0}{2-\mu\alpha_k\lambda_k{}^2)} > 0$, $\frac{\partial\pi_b^{BK}}{\partial\lambda_a} = \frac{2(1-\alpha_p)^2(1-\delta-\mu)^2\lambda_a}{2(2-\mu\alpha_k\lambda_k{}^2)^2} d_0{}^2 > 0$. Taking the first-order condition of e_r^{BK}, e_k^{BK}, s^{BK} and p^{BK} with respect to λ_k, we can get $\frac{\partial e_r^{BK}}{\partial\lambda_k} = \frac{-\delta\alpha_r\lambda_r d_0(-2\mu\alpha_k\lambda_k)}{(2-\mu\alpha_k\lambda_k{}^2)^2} > 0$, $\frac{\partial s^{BK}}{\partial\lambda_k} = \frac{-(1-\alpha_p)(1-\delta-\mu)\lambda_a d_0(-2\mu\alpha_k\lambda_k)}{(2n_k-\mu\alpha_k\lambda_k{}^2)^2} > 0$, $\frac{\partial e_k^{BK}}{\partial\lambda_k} = \frac{\mu\alpha_k d_0(2+\mu\alpha_k\lambda_k{}^2)}{(2-\mu\alpha_k\lambda_k{}^2)^2} > 0$, $\frac{\partial p^{BK}}{\partial\lambda_k} = \frac{-d_0(-2\mu\alpha_k\lambda_k)}{(2-\mu\alpha_k\lambda_k{}^2)^2} > 0$. Taking the first-order condition of π_b^{BK} with respect to λ_k, we can get $\frac{\partial\pi_b^{BK}}{\partial\lambda_k} = 2\mu\alpha_k\lambda_k d_0{}^2 \times \frac{2(1-\alpha_k-\alpha_p)\mu+2(1-\alpha_r-\alpha_p)\delta^2\alpha_r\lambda_r{}^2+(1-\alpha_p)^2(1-\delta-\mu)^2\lambda_a{}^2}{(2-\mu\alpha_k\lambda_k{}^2)^3} - 2\mu\alpha_k\lambda_k d_0{}^2 \times \frac{[(1-\alpha_r-\alpha_p)\delta+(1-\alpha_p)(1-\delta-\mu)]\mu\alpha_k\lambda_k{}^2}{(2-\mu\alpha_k\lambda_k{}^2)^3}$. The signs of $\frac{\partial\pi_b^{BK}}{\partial\lambda_k}$ depend on the numerator which increases in λ_k. It is to show that $\frac{\partial\pi_b^{BK}}{\partial\lambda_k} > 0$ for any $0 < \lambda_k < \sqrt{\frac{2(1-\alpha_k-\alpha_p)\mu+2(1-\alpha_r-\alpha_p)\delta^2\alpha_r\lambda_r{}^2+(1-\alpha_p)^2(1-\delta-\mu)^2\lambda_a{}^2}{[(1-\alpha_r-\alpha_p)\delta+(1-\alpha_p)(1-\delta-\mu)]\mu\alpha_k}}$. Taking the first-order condition of e_r^{PK} and π_b^{PK} with respect to λ_r, we can get $\frac{\partial e_r^{PK}}{\partial\lambda_r} = \frac{\delta\alpha_r d_0 n_k}{n_r(2n_k-\mu\alpha_k\lambda_k{}^2)} > 0$, $\frac{\partial\pi_b^{PK}}{\partial\lambda_r} = \frac{2(1-\alpha_r-\alpha_p)\delta^2\alpha_r\lambda_r n_k m}{n_r m(2n_k-\mu\alpha_k\lambda_k{}^2)^2} d_0{}^2 n_k > 0$. Taking the first-order condition of s^{PK} and π_b^{PK} with respect to λ_a, we can get $\frac{\partial s^{PK}}{\partial\lambda_a} = \frac{\alpha_p(1-\delta-\mu)d_0 n_k}{m(2n_k-\mu\alpha_k\lambda_k{}^2)}$, $\frac{\partial\pi_b^{PK}}{\partial\lambda_a} = \frac{2(1-\alpha_p)(1-\delta-\mu)^2\alpha_p\lambda_a n_r n_k}{n_r m(2n_k-\mu\alpha_k\lambda_k{}^2)^2} d_0{}^2 n_k > 0$. Taking the first-order condition of e_r^{PK}, e_k^{PK}, s^{PK} and p^{PK} with respect to λ_k, we can get $\frac{\partial e_r^{PK}}{\partial\lambda_k} = \frac{-\delta\alpha_r\lambda_r d_0 n_k n_r(-2\mu\alpha_k\lambda_k)}{[n_r(2n_k-\mu\alpha_k\lambda_k{}^2)]^2} > 0$, $\frac{\partial s^{PK}}{\partial\lambda_k} = \frac{-\alpha_p(1-\delta-\mu)\lambda_a d_0 n_k m(-2\mu\alpha_k\lambda_k)}{[m(2n_k-\mu\alpha_k\lambda_k{}^2)]^2} > 0$, $\frac{\partial e_k^{PK}}{\partial\lambda_k} = \frac{\mu\alpha_k d_0(2n_k+\mu\alpha_k\lambda_k{}^2)}{(2n_k-\mu\alpha_k\lambda_k{}^2)^2} > 0$, $\frac{\partial p^{PK}}{\partial\lambda_k} = \frac{-d_0 n_k(-2\mu\alpha_k\lambda_k)}{(2n_k-\mu\alpha_k\lambda_k{}^2)^2} > 0$. Taking the first-order condition of π_b^{PK} with respect to λ_k, we can get $\frac{\partial\pi_b^{PK}}{\partial\lambda_k} = 2\mu\alpha_k\lambda_k d_0{}^2 \times \frac{2(1-\alpha_k-\alpha_p)\mu+2(1-\alpha_r-\alpha_p)\delta^2\alpha_r\lambda_r{}^2+2(1-\alpha_p)(1-\delta-\mu)^2\alpha_p\lambda_a{}^2}{(2-\mu\alpha_k\lambda_k{}^2)^3} - 2\mu\alpha_k\lambda_k d_0{}^2 \times \frac{[(1-\alpha_r-\alpha_p)\delta+(1-\alpha_p)(1-\delta-\mu)]\mu\alpha_k\lambda_k{}^2}{(2-\mu\alpha_k\lambda_k{}^2)^3}$. The signs of $\frac{\partial\pi_b^{PK}}{\partial\lambda_k}$ depend on the numerator which increases in λ_k. It is easy to show that $\frac{\partial\pi_b^{PK}}{\partial\lambda_k} > 0$ for any $0 < \lambda_k < \sqrt{\frac{2[(1-\alpha_k-\alpha_p)\mu+(1-\alpha_r-\alpha_p)\delta^2\alpha_r\lambda_r{}^2+(1-\alpha_p)(1-\delta-\mu)^2\alpha_p\lambda_a{}^2]}{[(1-\alpha_r-\alpha_p)\delta+(1-\alpha_p)(1-\delta-\mu)]\mu\alpha_k}}$. □

Proof to Corollary 4: Taking the first-order condition of e_r^{BK}, e_r^{PK}, s^{BK} and s^{PK} with respect to δ, we can get $\frac{\partial e_r^{BK}}{\partial\delta} = \frac{\alpha_r\lambda_r d_0 n_k}{n_r(2n_k-\mu\alpha_k\lambda_k{}^2)} > 0$, $\frac{\partial e_r^{PK}}{\partial\delta} = \frac{\alpha_r\lambda_r d_0 n_k}{n_r(2n_k-\mu\alpha_k\lambda_k{}^2)} > 0$, $\frac{\partial s^{BK}}{\partial\delta} = \frac{-(1-\alpha_p)\lambda_a d_0 n_k}{m(2n_k-\mu\alpha_k\lambda_k{}^2)} < 0$, $\frac{\partial s^{PK}}{\partial\delta} = \frac{-\alpha_p\lambda_a d_0 n_k}{m(2n_k-\mu\alpha_k\lambda_k{}^2)} < 0$. Taking the first-order condition of π_b^{BK} and π_b^{PK} with respect to δ, we

can get $\frac{\partial \pi_b^{BK}}{\partial \delta} = \frac{-\alpha_r + 2(1-\alpha_r-\alpha_p)\delta\alpha_r{\lambda_r}^2 - (1-\alpha_p)^2(1-\delta-\mu){\lambda_a}^2 + \mu\alpha_r\alpha_k{\lambda_k}^2}{(2-\mu\alpha_k{\lambda_k}^2)^2}{d_0}^2$, $\frac{\partial \pi_b^{PK}}{\partial \delta} = \frac{-\alpha_r + 2(1-\alpha_r-\alpha_p)\delta\alpha_r{\lambda_r}^2 - 2\alpha_p(1-\alpha_p)(1-\delta-\mu){\lambda_a}^2 + \mu\alpha_r\alpha_k{\lambda_k}^2}{(2-\mu\alpha_k{\lambda_k}^2)^2}{d_0}^2$. The signs of $\frac{\partial \pi_b^{BK}}{\partial \delta}$ and $\frac{\partial \pi_b^{PK}}{\partial \delta}$ depend on the numerator which increases in λ_r and λ_k. It is easy to show that $\frac{\partial \pi_b^{BK}}{\partial \delta} < 0$ for any $0 < \lambda_r < \sqrt{\frac{\alpha_r + (1-\alpha_p)^2(1-\delta-\mu){\lambda_a}^2 - \mu\alpha_r\alpha_k{\lambda_k}^2}{2(1-\alpha_r-\alpha_p)\delta\alpha_r}}$ and $0 < \lambda_k < \sqrt{\frac{\alpha_r + (1-\alpha_p)^2(1-\delta-\mu){\lambda_a}^2}{\mu\alpha_r\alpha_k}}$. The same is easy to show that $\frac{\partial \pi_b^{PK}}{\partial \delta} < 0$ for any $0 < \lambda_r < \sqrt{\frac{\alpha_r + 2(1-\alpha_p)(1-\delta-\mu)\alpha_p{\lambda_a}^2 - \mu\alpha_r\alpha_k{\lambda_k}^2}{2(1-\alpha_r-\alpha_p)\delta\alpha_r}}$ and $0 < \lambda_k < \sqrt{\frac{\alpha_r + 2(1-\alpha_p)(1-\delta-\mu)\alpha_p{\lambda_a}^2}{\mu\alpha_r\alpha_k}}$. Taking the first-order condition of e_r^{BK}, e_r^{PK}, e_k^{BK}, e_k^{PK}, p^{BK} and p^{PK} with respect to μ, we can get $\frac{\partial e_r^{BK}}{\partial \mu} = \frac{\delta\alpha_r\lambda_r\alpha_k{\lambda_k}^2 d_0}{(2-\mu\alpha_k{\lambda_k}^2)^2} > 0$, $\frac{\partial e_r^{PK}}{\partial \mu} = \frac{\delta\alpha_r\lambda_r\alpha_k{\lambda_k}^2 d_0}{(2-\mu\alpha_k{\lambda_k}^2)^2} > 0$, $\frac{\partial e_k^{BK}}{\partial \mu} = \frac{2\alpha_k\lambda_k d_0}{(2-\mu\alpha_k{\lambda_k}^2)^2} > 0$, $\frac{\partial e_k^{PK}}{\partial \mu} = \frac{2\alpha_k\lambda_k d_0}{(2-\mu\alpha_k{\lambda_k}^2)^2} > 0$, $\frac{\partial p^{BK}}{\partial \mu} = \frac{-d_0(-\alpha_k{\lambda_k}^2)}{(2-\mu\alpha_k{\lambda_k}^2)^2} > 0$, $\frac{\partial p^{PK}}{\partial \mu} = \frac{\alpha_k{\lambda_k}^2 d_0}{(2-\mu\alpha_k{\lambda_k}^2)^2} > 0$. Taking the first-order condition of s^{BK} and s^{PK} with respect to μ, we can get $\frac{\partial s^{BK}}{\partial \mu} = \frac{(1-\alpha_p)\lambda_a d_0[(1-\delta)\alpha_k{\lambda_k}^2 - 2]}{(2-\mu\alpha_k{\lambda_k}^2)^2}$ and $\frac{\partial s^{PK}}{\partial \mu} = \frac{\alpha_p\lambda_a d_0[(1-\delta)\alpha_k{\lambda_k}^2 - 2]}{(2-\mu\alpha_k{\lambda_k}^2)^2}$. The signs of $\frac{\partial s^{BK}}{\partial \mu}$ and $\frac{\partial s^{PK}}{\partial \mu}$ depend on the numerator which increases in λ_k. It is easy to show that $\frac{\partial s^{BK}}{\partial \mu} < 0$ and $\frac{\partial s^{PK}}{\partial \mu} < 0$ for any $0 < \lambda_k < \sqrt{\frac{2}{(1-\delta)\alpha_k}}$. □
